# Top abundant deep ocean heterotrophic bacteria can be retrieved by cultivation

**DOI:** 10.1038/s43705-023-00290-0

**Published:** 2023-09-02

**Authors:** Isabel Sanz-Sáez, Pablo Sánchez, Guillem Salazar, Shinichi Sunagawa, Colomban de Vargas, Chris Bowler, Matthew B. Sullivan, Patrick Wincker, Eric Karsenti, Carlos Pedrós-Alió, Susana Agustí, Takashi Gojobori, Carlos M. Duarte, Josep M. Gasol, Olga Sánchez, Silvia G. Acinas

**Affiliations:** 1 Departament de Biologia Marina i Oceanografia, Institut de Ciències del Mar, ICM-CSIC, 08003 Barcelona, Spain; 2 Department of Biology, Institute of Microbiology, ETH Zurich, Vladimir-Prelog-Weg 1-5/10, CH-8093 Zurich, Switzerland; 3 Sorbonne University, CNRS, Station Biologique de Roscoff, UMR7144, ECOMAP, Roscoff, France; 4 Institut de Biologie de l’École Normale Supérieure (IBENS), École Normale supérieure, CNRS, INSERM, PSL Université Paris, 75005 Paris, France; 5 Departments of Microbiology and Civil, Environmental and Geodetic Engineering; The Ohio State University, Columbus, OH 43210 USA; 6 Génomique Métabolique, Genoscope, Institut de Biologie François Jacob, Commissariat à l’Énergie Atomique (CEA), CNRS, Université Evry, Université Paris-Saclay, 91000 Evry, France; 7 Research Federation for the Study of Global Ocean Systems Ecology and Evolution, FR2022/Tara Oceans GOSEE, 75016 Paris, France; 8 Directors’ Research European Molecular Biology Laboratory, 69117 Heidelberg, Germany; 9 Department of Systems Biology, Centro Nacional de Biotecnología (CNB), CSIC, 28049 Madrid, Spain; 10 Red Sea Research Center, King Abdullah University of Science and Technology (KAUST), Thuwal, 23955-6900 Saudi Arabia; 11 Computational Bioscience Research Center (CBRC), King Abdullah University of Science and Technology (KAUST), Thuwal, 23955-6900 Saudi Arabia; 12 Departament de Genètica i Microbiologia, Facultat de Biociències, Universitat Autònoma de Barcelona, 08193 Bellaterra, Spain

**Keywords:** Microbial ecology, Environmental microbiology

## Abstract

Traditional culture techniques usually retrieve a small fraction of the marine microbial diversity, which mainly belong to the so-called rare biosphere. However, this paradigm has not been fully tested at a broad scale, especially in the deep ocean. Here, we examined the fraction of heterotrophic bacterial communities in photic and deep ocean layers that could be recovered by culture-dependent techniques at a large scale. We compared 16S rRNA gene sequences from a collection of 2003 cultured heterotrophic marine bacteria with global 16S rRNA metabarcoding datasets (16S TAGs) covering surface, mesopelagic and bathypelagic ocean samples that included 16 of the 23 samples used for isolation. These global datasets represent 60 322 unique 16S amplicon sequence variants (ASVs). Our results reveal a significantly higher proportion of isolates identical to ASVs in deeper ocean layers reaching up to 28% of the 16S TAGs of the bathypelagic microbial communities, which included the isolation of 3 of the top 10 most abundant 16S ASVs in the global bathypelagic ocean, related to the genera *Sulfitobacter*, *Halomonas* and *Erythrobacter*. These isolates contributed differently to the prokaryotic communities across different plankton size fractions, recruiting between 38% in the free-living fraction (0.2–0.8 µm) and up to 45% in the largest particles (20–200 µm) in the bathypelagic ocean. Our findings support the hypothesis that sinking particles in the bathypelagic act as resource-rich habitats, suitable for the growth of heterotrophic bacteria with a copiotroph lifestyle that can be cultured, and that these cultivable bacteria can also thrive as free-living bacteria.

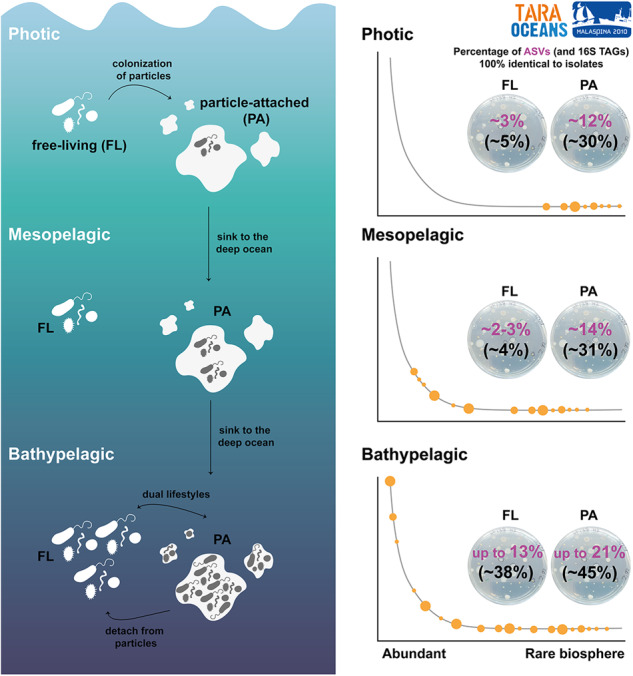

## Introduction

Isolation of bacteria in pure culture is challenging. Traditionally, it has been said that only a small fraction of the natural bacterial communities can be cultivated, a phenomenon that has been called “the great plate count anomaly” [1]. The recovered proportion of cells using selective media and standard plating techniques when compared to microscopy counts by direct staining is thought to represent only among 0.001–1% of the community [1–4]. This phenomenon led to the known paradigm that “less than 1% of the microbial cells can be cultured” [1, 5–7]. In fact, most of the marine bacterial strains growing under laboratory conditions belong to the rare biosphere [8, 9] with some key exceptions such as *Prochlorococcus* and *Synechococcus*, which represent the most abundant and widespread phytoplankton taxa in the global ocean [10–12], and the most abundant heterotrophic bacteria in the surface ocean like SAR11 isolated using high-throughput dilution-to-extinction culture techniques [13–18]. Moreover, when targeting the marine heterotrophic culturable bacteria from marine ecosystems, most of the studies have focused on the upper ocean (0–200 m depth) or on specific oceanographic regions [16, 19–21], while studies covering different depths are less frequent [22–24]. Efforts to culture bacteria from the deep ocean (>200 m) have focused mostly on isolates from hydrothermal vents [25–27], whale carcasses [28], trenches [29], and deep-sea sediments [30–33]. However, very few studies have attempted to isolate bacteria from the mesopelagic [34–36], or the bathypelagic waters [23, 37–39], and those available were mainly done at a local or regional scale but not a large scale. Therefore, the long-standing observation that traditional culture techniques only retrieve a small fraction of the microbial diversity in marine environments still needs to be properly tested in the pelagic deep ocean.

On the other hand, the overlap between isolated microorganisms and those belonging to the uncultured majority are relatively low as proved by various studies comparing culture-dependent and culture-independent techniques in marine ecosystems [18, 21, 40–42]. Besides, some meta-analysis studies have analyzed both the abundance and the diversity of the prokaryotic community that can be isolated in different ecosystems, including marine environments [43–45]. However, the deep ocean was poorly examined, and the authors used different genetic thresholds and methods for calculating which percentage of the prokaryotic diversity could be isolated generating contrasting results. Most importantly, they did not compare isolates and 16S amplicon TAGs from exactly the same samples. Hence, the aim of our study, far from isolating and describing novel bacteria, was to use well established marine solid media to retrieve the fraction of the bacterioplankton community than can be commonly isolated under laboratory conditions (nutrient rich medium, standard oxygen concentrations and atmospheric pressure), combining both culture-dependent and -independent techniques including the photic and the deep ocean from both mesopelagic and bathypelagic zones in order to be able to compare results across different oceanographic regions and depths.

Diversity of microbial communities from the bathypelagic ocean across the tropical and temperate oceans had described the most abundant operational taxonomic units (OTUs) [46] and interestingly, some of these OTUs affiliated with well-known heterotrophic bacterial genera that can be easily retrievable in culture, including *Alteromonas*, *Alcanivorax* and *Halomonas* [23, 46, 47]. Some of these deep-ocean marine bacteria, such as *Alteromonas*, are well-known copiotrophs [48] and given that the bathypelagic realm is fed by sinking particles [49], which are likely resource-rich habitats for microbes [50, 51], and that has recently been proven that a higher proportion of bacteria have been isolated from particles compared to free-living communities [52], we hypothesize that: i) by using traditional culture-dependent techniques a high proportion of the bacteria dwelling in the deep ocean would be retrieved under laboratory conditions, and ii) particles in the deep ocean are hotspots of copiotrophic bacteria that may be more easily isolated in culture than the free-living ones, or than those present at the surface.

## Results

**Fig. 1:**
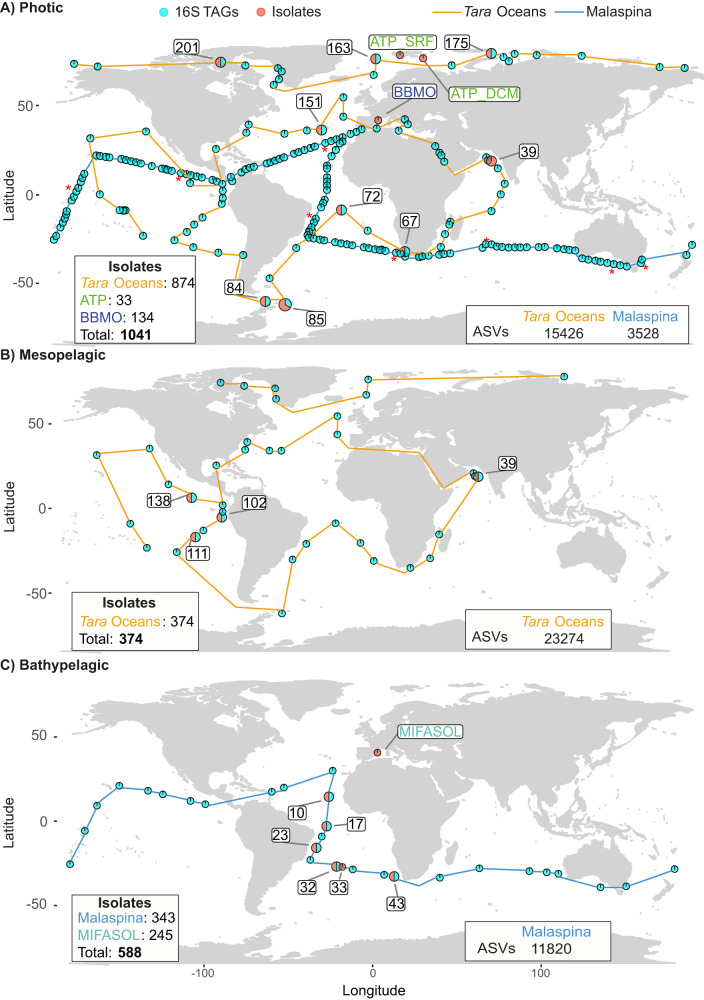
World map showing the distribution of the samples used in this study per ocean layer. **A** Photic. Labeled samples correspond to those stations where isolates were obtained from: *Tara* Oceans (39, 67, 72, 76, 84, 85, 151, 163, 175, 201), Blanes Bay Microbial Observatory (BBMO), and ATP Arctic cruise (ATP_SRF, ATP_DCM). Stations with red asterisks correspond to amplicon 16S TAGs from eight vertical profiles with five different microbial size fractions collected from the Malaspina Expedition. **B** Mesopelagic. Labeled samples correspond to those stations where isolates were obtained from the *Tara* Oceans (39, 102, 111, 138). **C** Bathypelagic. Labeled samples correspond to those stations where isolates were obtained from: Malaspina (10, 17, 23, 32, 43) and MIFASOL. Circles connected with a blue line show the distribution of the samples obtained from the Malaspina Expedition, while circles connected with an orange line show those from the *Tara* Oceans. Each pie chart shows the presence or absence of samples from the different datasets: orange, isolates; and light-blue, metabarcoding 16S TAGs.

We examined the fraction of heterotrophic microbial communities that could be retrieved by isolation from both the photic and the deep ocean, and explored how abundant are cultured bacteria in different plankton size fractions. To that end, we combined results from a large collection of heterotrophic cultured bacteria (MARINHET_v2), covering a wide range of oceanographic regions and depths, including the photic, the mesopelagic and the bathypelagic ocean [47], with culture-independent results including flow cytometry measurements and 16S metabarcoding datasets obtained from simultaneous samples as the ones used for isolation from global oceanographic expeditions, *Tara* Oceans 2009–2013 (including *Tara* Oceans Polar Circle samples) [53] and Malaspina Circumnavigation Expedition 2010 [54]. Details regarding all samples used in this study can be found in Fig. 1.

### Description of the MARINHET_v2 culture collection

**Fig. 2:**
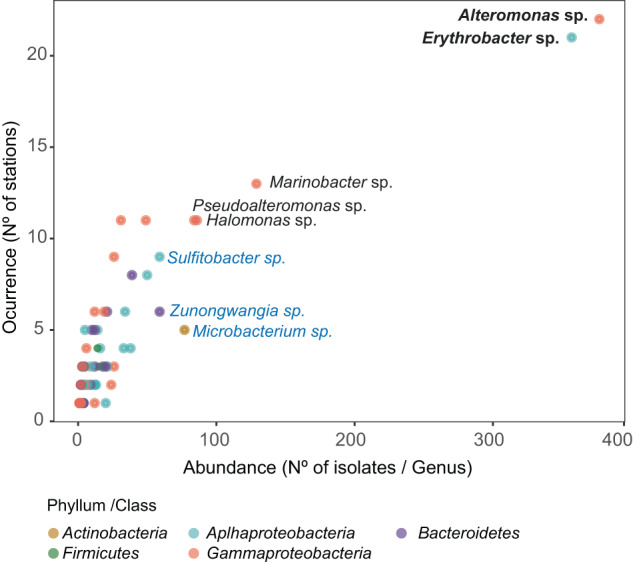
Abundance versus occurrence of the genera retrieved in the total culture collection. Taxonomic classification is indicated for those genera presenting more than 50 isolates in the whole culture collection. The most commonly retrieved genera (>80% of the samples) are indicated in bold. Those genera present in 50% of the samples studied are indicated in black letters, while those appearing in blue represent those genera with a more regional distribution (>25% of the samples). The color of the dots indicates the taxonomic (phylum or class) affiliation of the genera.

A total of 2003 isolates were retrieved from 23 marine stations, twelve photic-layer (1041 isolates), four mesopelagic (374 isolates) and seven bathypelagic (588 isolates) stations (Fig. 1) affiliating to 90 different genera (35 *Alphaproteobacteria*, 26 *Gammaproteobacteria*, 17 *Bacteroidetes*, 10 *Actinobacteria*, and 2 *Firmicutes*). Extensive description of the MARINHET culture collection including 1561 isolates of the 2003 presented in this study has been previously described [47] including diversity metrices, taxonomic and phylogenetic description, as well as, biogeography of the cultured bacteria. Therefore, in order to confirm some of the patterns observed previously, rarefaction and accumulation curves were performed with the isolates clustered at 100% (isolates OTUs) (Supplementary Fig. S1), and results indicated that the isolates dataset, even if not saturated, represents a reasonable inventory of the culturable heterotrophic marine bacteria. In addition, biogeography of the cultured dataset was explored (Fig. 2) in order to detect how many genera occurred in all or most of the 23 stations studied. Thus, *Alteromonas sp*. and *Erythrobacter* sp. were the most abundant and recurrent genera regardless of the oceanographic region or depth as they were isolated in more than 80% of the stations; five genera including *Marinobacter* sp., *Halomonas* sp., *Idiomarina* sp., *Pseudoalteromonas* sp. and *Pseudomonas* sp. were isolated in 50% of the samples, nine other genera were retrieved regionally (>25% of the samples), while the remaining cultured genera presented a local distribution (<25% of the samples) (Supplementary Table S1).

### Testing the “great plate count anomaly” in different oceanic regions and depths

**Fig. 3:**
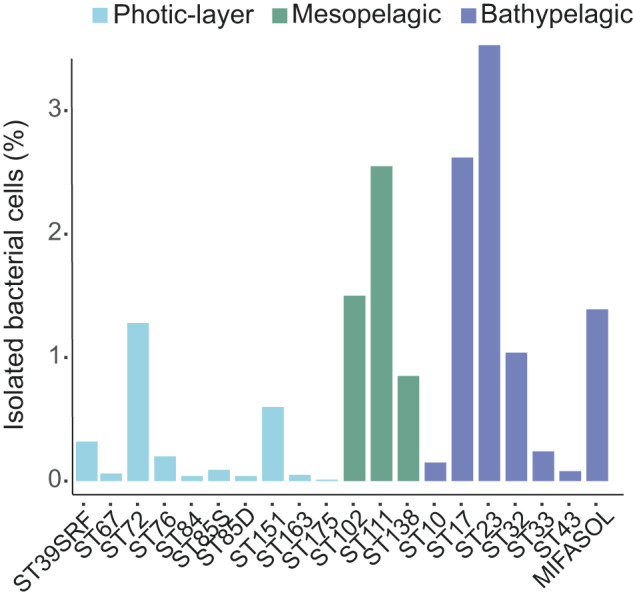
Testing the great plate count anomaly in different oceanic regions and ocean layers. For each station, heterotrophic prokaryotic abundances were estimated using both, traditional culture techniques (cfu/ml) and flow cytometry (cells/ml). The percentages represent the fraction of heterotrophic prokaryote abundance that could be retrieved by culturing in photic, mesopelagic and bathypelagic stations. Color indicates the depth of the sample: cyan, photic-layer; turquoise, mesopelagic; and dark-blue, bathypelagic. No significative differences were found between layers (ANOVA, *P*-value > 0.05).

We calculated the percentage of isolated bacterial cells for ten photic-layer, three mesopelagic and seven bathypelagic stations where plate colony counts (cfu/ml) and flow cytometry values -as a measure of total prokaryotic abundance/concentration- (cells/ml) were available (Fig. 3). For this comparative analysis, flow cytometry counts included only the abundance of all heterotrophic prokaryotes and excluded photosynthetic Cyanobacteria since these taxa were not targeted by the media nor incubation conditions selected. Considering that, we detected a higher percentage of recovery in the mesopelagic and bathypelagic samples (1.3%) compared to the photic layer (0.3%), although the differences were not significant (ANOVA, *P*-value > 0.05). The percentage of cultivability of heterotrophic bacterial cells ranged from 0.01 to 1.3% in photic-layer samples, and from 0.9 to 2.5% in mesopelagic samples, while percentages for bathypelagic samples varied between 0.08% and 3.5%, indicating a higher success in isolation in some samples from the deeper layers of the ocean (Fig. 3).

### Contribution of culturable bacteria to total prokaryotic diversity in different ocean layers

The MARINHET_v2 culture collection was compared with two global metabarcoding datasets (16S TAGs) amplified with the same primer set (515F-Y-926R) [55] with a total of 38 700 ASVs from *Tara* Oceans (15 426 *Tara Oceans* Surface and 23 274 *Tara Oceans* Mesopelagic) and 15 348 ASVs from the Malaspina Expedition (3528 Malaspina Surface and 11 820 Malaspina Bathypelagic) (Supplementary Table S2, Fig. 1). We determined the mean percentage of ASVs (diversity) as well as the mean percentage of 16S TAGs (abundance) that were 100% identical to our MARINHET_v2 culture collection isolates. A summary of the results obtained from the rarefied ASVs-abundance tables is shown in Supplementary Table S3.

**Fig. 4:**
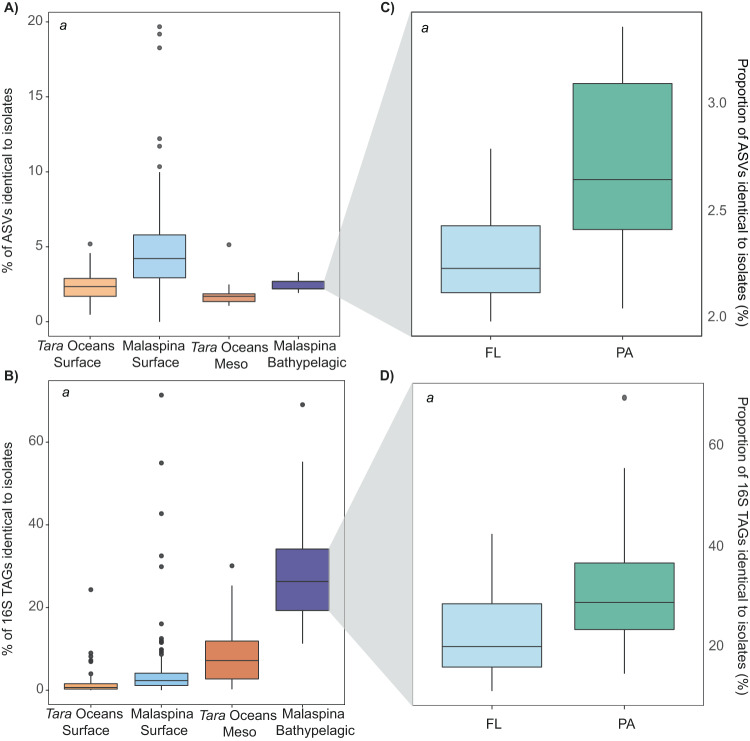
Summary of the percentages of ASVs or 16S TAGs identical to isolates in the datasets analyzed. **A** Proportion of isolates matching at 100% identity to ASVs. **B** Proportion of 16S TAGs reads matching isolates at 100 % similarity. Values are extracted from the mean abundance of reads or ASVs in each dataset from rarified ASV-abundance tables. Outliers are indicated with gray circles. If significant differences are found between all datasets it is indicated inside boxplots with an italic *a* (Kruskal–Wallis, *P*-value < 2.2e−16). **C** Proportion of isolates that are 100% similar to ASVs when separating free-living and particle-attached size fractions in the Malaspina Bathypelagic dataset. **D** Proportion of 16S TAGs reads matching isolates at 100% similarity when separating free-living and particle-attached size fractions in the Malaspina Bathypelagic dataset. Significative differences between size fractions (*P*-value < 0.01) are indicated by an italic *a* in the top left corner.

The highest average number of ASVs that were 100% identical to the isolates was observed in the Malaspina Surface dataset (4.5%), followed by the Malaspina Bathypelagic (2.4%), *Tara* Oceans Surface (2.3%) and *Tara* Oceans Mesopelagic datasets (1.7%) (Fig. 4A). Even though these percentages do not seem to vary greatly between datasets, significant differences were found among them (Kruskal–Wallis, *P*-value < 0.01, Fig. 4A). Otherwise, if we consider the abundances of these ASVs and we look at the percentage of reads (16S TAGs) identical to the isolates, we observed a significant increase in the deep ocean (Fig. 4B). Thus, around 1.6–4.9% of the 16S TAGs were 100% identical to our isolates in the photic ocean, this value increased up to 8.5% in the mesopelagic ocean, and increased even more, up to 27.9%, in the bathypelagic ocean. In this case, the differences between datasets were also statistically significant (Kruskal–Wallis test, *P*-value < 0.01, Fig. 4B). The metabarcoding 16S TAGs dataset of Malaspina Bathypelagic samples integrated free-living (0.2–0.8 µm) and particle-attached (0.8–20 µm) microbial communities. However, if we only used the data from the free-living fraction (to be fair with the comparisons with the free-living bacteria analyzed from the photic and mesopelagic samples), we still observed this trait of higher proportions, with 22.9% of the 16S TAGs 100% identical to isolates in the bathypelagic samples (Fig. 4C, D). Comparisons where photic and aphotic zone isolates are compared separately with all datasets is shown in Supplementary Fig. S2. The same pattern was noticed and we detected a higher proportion of ASVs and 16S TAGs identical to isolates when comparing aphotic zone isolates only.

A fraction of the isolates did not match any ASV regardless of the dataset inspected (Supplementary Table S4). Indeed, approximately 11% of our heterotrophic isolates did not match any of the *Tara* Oceans ASVs, whereas this number increased up to 18% in the Malaspina Bathypelagic ASVs and up to 28% in the surface samples from Malaspina (Supplementary Fig. S3). Some interesting taxonomic differences at the family level were observed between the isolates that were identical to ASVs and those that did not match any ASV (Supplementary Figs. S4 and S5). We found some families that were identified in the *Tara* Oceans Surface and Mesopelagic metabarcoding datasets but not in the Malaspina Expedition Surface or Bathypelagic metabarcoding datasets, such as *Tistrellaceae, Nitrincolaceae or Colwelliaceae*. In contrast, *Kangiellaceae* was found in both Malaspina Expedition datasets but not in *Tara* Oceans samples. Some families included isolates that did not match any ASVs in any of the 16S metabarcoding datasets, such as *Dermabacteraceae*, *Balneolaceae* or *Psychromonadaceae* (Supplementary Figs. S4 and S5). Within those families not detected by our sequencing data, we found some genera such as *Balneola* sp., *Nereida* sp., *Ruegeria* sp. or *Citreicella* sp. We tested if the primers used [55] presented a mismatch on their partial 16S rRNA sequences, but the primers could potentially capture also these organisms. All of these genera were only retrieved locally (<25% of the stations studied), indicating that they were present at very low abundances in the environment. Furthermore, based on the accumulation plots (Supplementary Fig. S6) of the 16S amplicon datasets, we can observe that the sequencing effort used in *Tara* Oceans (~5 × 10^5^ average reads per sample) and in the Malaspina Expedition samples (surface samples ~5.1 × 10^4^ average reads, bathypelagic ~9.7 × 10^5^ average reads) was satisfactory, reaching the *plateau* in most of the datasets except for the *Tara* Mesopelagic. Therefore, the percentages of 16S TAGs identical to isolates would remain more or less the same in all datasets. However, an increasing sequencing effort could help us to detect the proportion of the isolates which may belong to the extremely rare biosphere that could not be detected by this sequencing technique.

### Bacterial isolates among the most abundant ASVs of the deep ocean

**Fig. 5:**
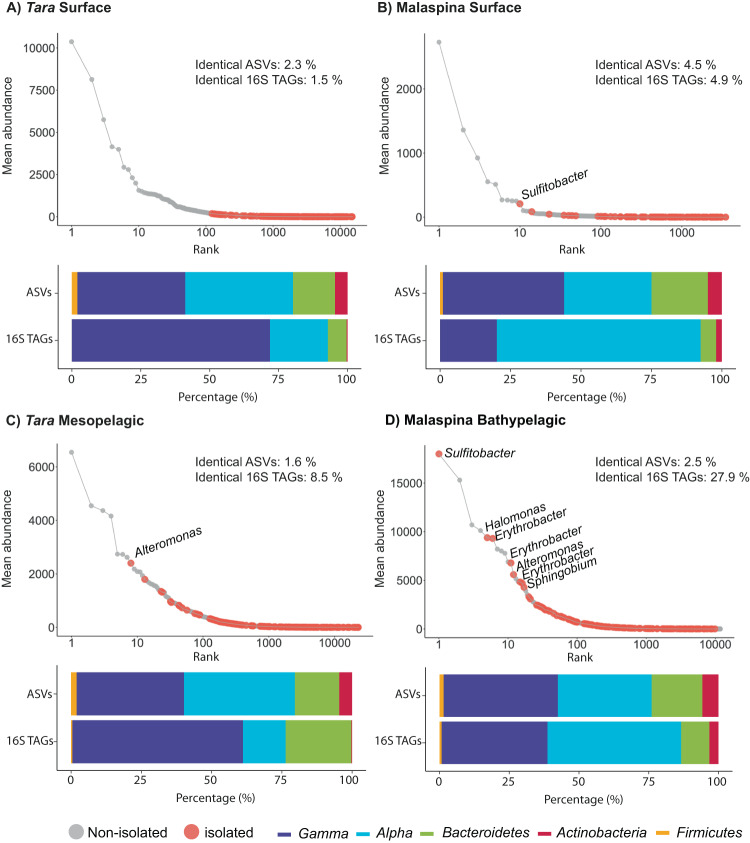
Rank plots showing the identified isolates that matched at 100% identity to 16S TAGs in different datasets. **A** *Tara* Oceans Surface. **B** Malaspina Surface. **C** *Tara* Oceans Mesopelagic. **D** Malaspina Bathypelagic. Gray dots: ASVs that did not match any isolate; orange dots, ASVs 100% identical to isolates. Taxonomic affiliation is indicated for the abundant (>1% abundance) ASVs that match with isolates. The histograms describe the proportions of ASVs or the proportion of 16S TAGs (reads) identical to isolates at the phylum/class level: dark-blue, *Gammaproteobacteria;* light-blue, *Alphaproteobacteria*; green, *Bacteroidetes*; red, *Actinobacteria*; and orange, *Firmicutes*.

To test whether isolates belonged to the abundant or rare biosphere, rank abundance plots were done for each of the *Tara* Oceans and Malaspina Expedition metabarcoding datasets, with the ASVs mean abundances extracted from rarefied ASVs-abundance tables (Fig. 5). The rank abundances were plotted based on either the global comparison of all isolates (Fig. 5) or by comparing photic and aphotic zone isolates separately against photic 16S TAGs datasets, or photic and aphotic zone isolates separately versus mesopelagic and bathypelagic 16S TAGs datasets (Supplementary Fig. S7) giving similar results in all of them. Each rank abundance plot showed a similar pattern of few abundant ASVs (relative abundance >1%), relatively few mid-abundant ASVs (<1% and >0.01%) and a long tail of rare or low-abundant ASVs (relative abundance <0.01%). We colored the ASVs that were 100% similar to at least one isolate to test for differences between depths (Fig. 5). In photic layers, we did not have any isolate within the abundant taxa of the *Tara* Oceans Surface dataset (Fig. 5A), whereas only one belonging to the abundant biosphere was found in the Malaspina Surface dataset, taxonomically related to *Sulfitobacter* with 1.7% of the total reads or 16S TAGs (Fig. 5B). The rest of the isolates in these two large-scale photic datasets appeared in the mid-abundant biosphere (38 in Malaspina and three in *Tara* Oceans) or in the rare biosphere (54 in Malaspina and 151 in *Tara* Oceans). In the mesopelagic layer (*Tara* Oceans Mesopelagic) we only found one ASV identical to isolates classified into the abundant biosphere (associated with *Alteromonas* with 1.1% of the reads), although more isolates were identical to ASV with medium abundances (87 ASVs) (Fig. 5C). Interestingly, the most abundant bathypelagic ocean taxon according to the 16S TAGs matched at 100% identity with one of our isolates. This organism was related to *Sulfitobacter* and represented 4.6% of the reads. In total, seven ASVs identical to isolates belonged to the abundant biosphere in Malaspina Bathypelagic, affiliating with the genera *Sulfitobacter*, *Halomonas*, *Erythrobacter, Alteromonas and Sphingobium*, and the first three were included into the top 10 most abundant ASVs detected. These, together with a relatively large proportion of isolates that matched organisms of the mid-abundant biosphere (78 ASVs), and those matching within the rare biosphere (52 ASVs), recruited 28% of the environmental reads from the temperate and tropical global bathypelagic oceans (Fig. 5D). Thus, abundant ASVs could be retrieved by culture-dependent techniques, especially in the bathypelagic layer. Curiously, some of the most abundant ASVs in the bathypelagic dataset where also the most abundant genera retrieved in the culture collection. *Alteromonas* and *Erythrobacter* were the most commonly isolated genera with more than 350 isolates recovered from more than 90% of the samples studied (Fig. 2), while *Sulfitobacter* sp. which is the most abundant ASVs of the Malaspina Bathypelagic dataset presented a regional distribution in our culture collection because it was retrieved from more than 25% of the samples but less than 50% of them. Interestingly, it was isolated from a higher number of aphotic stations (6 samples with 52 isolates in total) than photic stations (3 stations with 7 isolates) (Supplementary Table S1).

Altogether, we found that the ASVs that were 100% identical to our isolates affiliated mostly with classes *Alphaproteobacteria* (average 39% ASVs) and *Gammaproteobacteria* (average 47.7% ASVs) followed by phyla *Bacteroidetes* (average 11.5%), *Actinobacteria* (average 1.5%) and *Firmicutes* (average 0.3%) (Fig. 5 and Supplementary Table S5). We noticed that despite finding relatively similar proportions of isolates belonging to *Alphaproteobacteria* and *Gammaproteobacteria* in all ocean layers, the proportion of reads within these isolates identical to ASVs of these classes differed between the *Tara* Oceans and Malaspina Expedition datasets regardless of the sampling depth. In *Tara* Oceans samples, *Gammaproteobacteria* dominated (72%–61%), while *Alphaproteobacteria* dominated in the Malaspina datasets (72%–48%) (Fig. 5). These differences could be explained partially due to the different ocean regions, latitudes and seasons sampled by each expedition. Thus, more coastal regions were sampled in *Tara* Oceans, and the Malaspina Expedition reached deeper layers (up to 4000 m depth) compared to *Tara* Oceans.

### Increase of cultured isolates sequences at larger plankton size fractions

We also aimed to elucidate whether a higher proportion of isolates could be retrieved from bacteria developing on particles (PA: particle-attached bacteria) in the deep ocean representing hotspots of copiotrophic bacteria than those living as free-living bacteria (FL). We observed a significant increase (Wilcoxon test, *P*-value < 0.01) of isolates 100% identical to ASVs and of reads in the PA communities (0.8–20 µm, average 32.6%, sd:13.3%) versus the FL fraction (0.2–0.8 µm, average 22.99%, sd: 10.2%) (Fig. 4C, D) in the Malaspina Bathypelagic samples. However, the PA community analyzed in the Malaspina Bathypelagic dataset corresponded to particles of many different sizes from 0.8 to 20 µm plankton size fraction. Therefore, to explore the effect of the particle size range on the percentage of ASVs recovery, we also compared our isolates with samples from five different plankton size fractions (0.2–0.8 µm -considered FL; or PA in sizes: 0.8–3.0 µm, 3.0–5.0 µm, 5.0–20 µm, and 20–200 µm) in eight vertical profiles from the Malaspina Expedition.

**Fig. 6:**
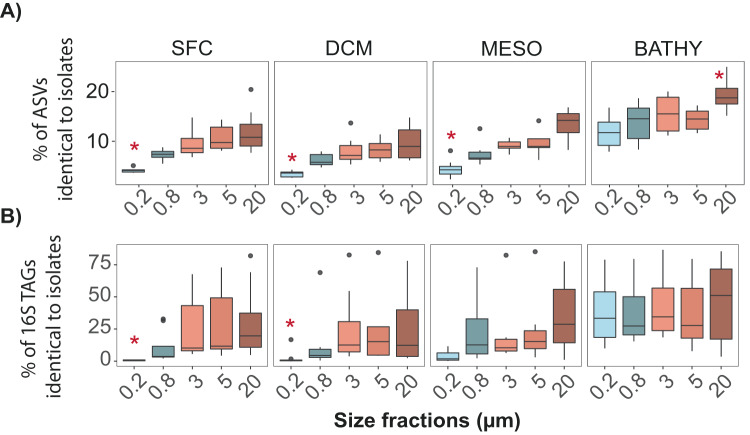
Boxplots comparing five different plankton size fractions in the Malaspina Expedition vertical profiles. **A** Percentage of isolates identical to ASVs per size fraction and depth. **B** Percentage of reads (16S TAGs) that were 100% identical to at least one isolate. SFC surface, DCM deep chlorophyll maximum, MESO mesopelagic, and BATHY bathypelagic. 0.2 μm: free-living bacteria (0.2–0.8 µm), 0.8 μm: bacteria attached to small particles (0.8–3 µm), and 3.0–5.0 and 5.0-20 μm: bacteria attached to larger particles. Significant differences between size fractions in each layer are indicated by red asterisks (Kruskal–Wallis, *P*-value < 0.05).

Our analysis revealed that the isolates were present across all size fractions and depths, yet the proportions varied (Fig. 6 and Supplementary Fig. S8). First, when looking at the differences between layers (surface, DCM, mesopelagic and bathypelagic), we confirmed again the highest average diversity (% of ASVs) and abundance (% of 16S TAGs) of isolated bacteria in the bathypelagic. The 16.3% of ASVs 100% identical to isolates from the bathypelagic (Fig. 6A) recruited an average of 40% of 16S TAGs (Fig. 6B). Among surface, DCM and mesopelagic samples, a similar number of isolates were detected (∼8% average) and also similar proportions of identical 16S TAGs were identified (∼20% average) (Fig. 6). Interestingly, the DCM was the layer with the smallest number of ASVs identical to isolates (~6% average) and 16S TAGs recruited (~17.3% on average). Given the notable proportion of ASVs identical to isolates in the bathypelagic samples, statistically significant differences were found between this deeper layer and the other depths in the different size fractions (Kruskal–Wallis test, *P*-values < 0.01), but not between surface, DCM or mesopelagic samples (Supplementary Figs. S8 and S9).

Additionally, the proportion of sequences identical to isolates across different plankton size fractions, revealed that our isolates were prevalent in the larger plankton size fractions, those associated with particles (≥3.0 μm) (Fig. 6). In samples from the photic zone (surface and DCM) higher mean abundances of ASVs and 16S TAGs (10% and 26% on average, respectively) were recovered by isolation in the largest size fractions (3.0 μm, 5.0 μm and 20 μm) but not in the FL bacterial communities (0.2 μm, 2.9% average ASVs, and 1.5% average 16S TAGs) or in the smallest particles (0.8 μm, 6% average ASVs, and 12% average 16S TAGs) (Supplementary Tables S6 and S7). In the mesopelagic samples, the number of ASVs identical to isolates (7%) in the 0.8 μm size fraction recruited up to 23% of the 16S TAGs, but not in the FL fraction (0.2 μm), which still presented lower values (4% of reads). Finally, in the bathypelagic samples, all plankton size fractions displayed similar percentages of ASVs identical to isolates, including the FL bacteria (average 16%), and uniform proportions of 16S TAGs (40% on average). In addition, the highest values were found from the largest particles (>20 μm, 21% of ASVs and 45% of the 16S TAGs).

## Discussion

Culturing studies are still fundamental for microbial ecologists to fully understand the physiology and ecology of microorganisms in marine ecosystems and test hypothesis that with sequencing techniques alone cannot be fully answered. In this study, combination of culture-dependent and culture-independent techniques have allowed us to test the great plate count anomaly paradigm in the deep ocean including mesopelagic and bathypelagic zones, and to test the hypothesis weather bathypelagic bacteria and those bacteria associated to particles are more prone to be isolated in pure culture.

The precise meaning of the traditional paradigm that only 1% of microbes are culturable has a difficult interpretation as discussed in Martiny’s study (2019) [45]. Our comparative analyses between the number of colonies retrieved in pure culture and the cell abundances calculated with flow cytometry provided information about the proportion of cells (abundance) than could be recovered by isolation using a specific set of culture and incubation conditions that were focused on the retrieval of commonly culturable heterotrophic bacteria but not in the discovery of novel microorganisms. Our results for mesopelagic and bathypelagic samples reflected a certain degree of variability, with a mean percentage of cultivability between 1.5% and 3.7%. This variability was highlighted in previous culture-dependent surveys covering a wide variety of environments: lakes, seawater, soils or sediments, and also human host-associated communities [43, 44]. However, none of these studies included open ocean samples deeper than 200 m. We can confirm that this variance can also be observed across the mesopelagic and the bathypelagic oceans, and that more than 1% of the cells and up to approximately 4% of them (Fig. 3) could be cultivated in some samples from the deep ocean. Therefore, the traditional paradigm that less than 1% of the microbial cells can be retrieved in culture still applies in the photic layers but should not be literally interpreted for the deep ocean. However, related but different questions are: which fraction of the microbial community diversity can be cultured?, and are the isolates abundant or rare members of the biosphere? To get these answers we compared the 16S rRNA sequences of the isolates at the maximal resolution possible (at 100% identity) with metabarcoding 16S TAGs from the same ocean samples.

The rank abundance curves of microbial communities are composed by some high-abundant and moderately abundant taxa but many low abundant taxa, the so called rare biosphere [56, 57]. We confirmed this structure for both expeditions datasets and different depth levels in the ocean, in agreement with previous studies [9, 46, 58, 59]. The common idea that cultivation mainly captures members of the rare biosphere seems to be the rule for bacteria in the photic layer, as the majority of the isolates from *Tara* Oceans and Malaspina (an average 81% isolates) recruited less than 1% of the total 16S rRNA sequences at 100% identity in datasets from the photic zone. These results were also observed in a single station in the northwestern Mediterranean Sea, in which 24% of the isolates from surface seawater were found in the amplicon data (454 TAGs), yet all belonged to rare taxa representing globally less than 1% of the total reads [9]. Similarly, a high-throughput sequencing study with >5000 bacterial strains isolated during a spring phytoplankton bloom in the North Sea pointed out that the 30 most frequently cultured operational phylogenetic units (OPUs) were represented with abundances below 0.6% in the 16S rRNA gene data [42].

More interestingly, we found that 2.4% of the isolates from the bathypelagic ocean recruited up to 28% of the reads of the total microbial community in the deep ocean realm identified by amplicon 16S TAGs (Fig. 4B) reflecting that we are capturing some of the abundant taxa of the deep ocean. Our isolation strategy was focused only on the heterotrophic marine bacteria and we are aware that the photosynthetic bacterial community in the surface samples is not captured with our isolation strategy nor are the Archaea, which could represent an important fraction of the bathypelagic ocean [46]. We tested what occurred when removing Cyanobacteria and Archaea TAGs from the datasets, and we obtained a similar trend with a slight increase, and identifying a similar average of the amplicon 16S TAGs reads identical to isolates in the surface, mesopelagic and bathypelagic datasets (Supplementary Table S8).

On the other hand, the 16S metabarcoding datasets used in this study are obtained with a PCR-dependent method, which could have influenced the results obtained given that isolated organisms usually have a higher rRNA operon copy number, and thus, overestimating the abundance of TAGs recruited by the ASVs identical to our isolates. This bias can be corrected by dividing our results by 3.5, which is the median rRNA operon copy number of the isolated genera in this study (Supplementary Table S9). Then, the proportion of TAGs recruited by ASV 100% identical to isolates is reduced in *Tara* Oceans Surface from 1.6% to 0.5%, in Malaspina Surface from 4.8% to 1.4%, in *Tara* Oceans Mesopelagic from 8.5% to 2.4% and in Malaspina Bathypelagic from 27.9% to 8 %. Even though the abundance of the isolated community diminishes, still higher proportions are found in the deeper layers and within those organisms we found some of the top 10 ASV detected among our culture collection, such as the most abundant ASV in the bathypelagic ocean affiliating to *Sulfitobacter* genera. *Sulfitobacter* is a well-recognized taxa involved in the degradation of the algal dimethylsulfoniopropionate (DMSP) [60], which is the major source of organic sulfur in the world’s oceans [61]. Other *Sulfitobacter* species, such as *Sulfitobacter* D7, can shift its lifestyle from coexistence to being pathogenic during its interaction with the bloom-forming phytoplankter *Emiliania huxleyi* [62], or *Sulfitobacter profundii* [63] isolated from deep ocean waters, which indicate the widespread distribution of this genus. Additionally, the closest relative based on the partial 16S rRNA gene is *Sulfitobacter pontiacus* [64] an ecologically relevant taxa involved in the sulfur cycle [65] and detected in different oceanographic regions and depths, including the deep ocean. Interestingly, we recovered one metagenome assembled genome (MAG) related to *Sulfitobacter pontiacus* (MAG0295) from our Deep Malaspina MAGs Dataset [66] reflecting that this genome is indeed abundant in the bathypelagic deep ocean, especially in some stations of the North Atlantic Ocean, and detected in both plankton size fractions (FL and PA, see Supplementary Fig. S10).

**Fig. 7:**
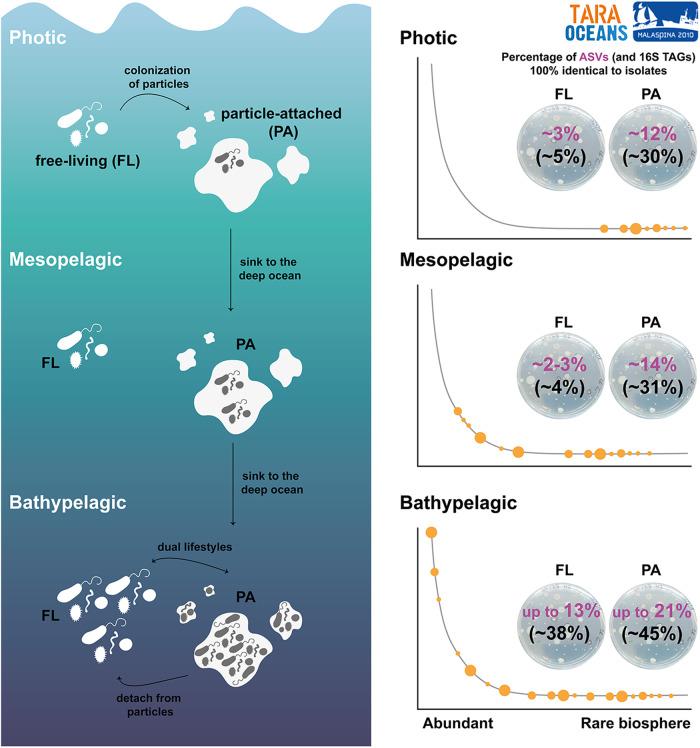
Conceptual representation of the heterotrophic culturable bacteria along the ocean water column. Free-living bacteria from photic ocean, which present a small fraction of heterotrophic culturable bacteria and mainly belonging to the rare biosphere, can attached to particles, where this culturable fraction is higher. These particles serve as a hotspot for growing and sink into the deep ocean where heterotrophic culturable bacteria become more abundant, specially in larger particles. Once in the deep ocean, mainly in the bathypelagic, bacteria can detach from particles and present a dual lifestyle. In the bathypelagic, some of the most abundant bacteria are culturable and they are present both in the free-living and in the particle-attached fraction.

Our research also highlights that the recruitment of isolates was higher in the particle-attached fraction of all layers compared to free-living communities, and especially in the largest size fractions of the bathypelagic ocean (Fig. 5B). These results are consistent with a recent study describing a larger cultivability from particle-attached communities from surface samples in the North Sea [52]. However, to the best of our knowledge, this is the first study to test the cultivability of communities attached to different size fractions in the deep ocean and in comparison with the photic layers. It is already well-know that marine microbial communities attached to particles are very different from the free-living ones both, taxonomically [67–71], and at the functional level [66, 72, 73]. A previous study that analyzed the same Malaspina size fractionated vertical profiles dataset [71] determined the importance of sinking particles as promoters of vertical connectivity in the ocean microbiome, and observed that bacteria associated with particles in the surface belonging to the rare taxa became dominant in the deep ocean. Our results for the culturable bacteria also confirmed that. Besides, the observed similarities in terms of proportion of 16S TAGs identical to isolates between size fractions in the bathypelagic ocean could be due to the presence of isolates with dual lifestyles (i.e. the same isolate is capable of living in particles and as a free-living bacteria), as has been reported for some marine *Flavobacteria* [74]. Additionally, a recent study on the globally active bathypelagic microbiome that combined 16S rRNA and 16S rDNA metabarcoding revealed the dominance of prokaryotes with dual lifestyles [75]. Furthermore, the isolates that were abundant (>1% relative abundance) in both free-living and particle-attached bacterial communities in the bathypelagic samples were also present in the largest size fractions of the surface layers, but appeared in the mid-abundant and rare biosphere of the free-living fraction (Supplementary Fig. S11 and Supplementary Table S10). Surface bacteria could thus act as a seed bank for bathypelagic communities, a hypothesis that has also been proposed in other studies [71, 76–78]. While our isolates likely belonged to the part of the bacterial community that prefers a particle-attached lifestyle, our results support the idea that they could live attached to particles in surface waters and sink with the particles to deeper layers where they would develop and finally become abundant members of the planktonic community (Fig. 7) with some of them presenting a dual lifestyle.

Particles are resource-rich habitats for microbes [50] where a copiotroph lifestyle could be the rule. Our findings support this theory given the fact that we found a higher proportion of isolates identical to the ASVs detected in the particle-attached communities compared to the free-living ones, especially in the bathypelagic communities. Also genomic comparisons between cultured isolates and uncultured genomes retrieved by single amplified genomes (SAGs) from marine environments showed that the genomes of the cultured bacteria had larger sizes, with a predominant copiotrophic lifestyle [79], which could have favored us in the recovery of the particle-attached bacteria using the culture and incubation conditions selected. Some of the most abundant ASVs identical to isolates detected in the bathypelagic ocean and affiliating to *Sulfitobacter*, *Halomonas* and *Alteromonas* display higher growth rates (>4 day^−1^) than other heterotrophic bacteria [80], which may also aid the isolation in pure culture. However, our MARINHET_v2 culture collection still misses most of the free-living bacteria adapted to oligotrophic conditions in the photic and the mesopelagic layers. Different reasons can be given to explain why still a high percentage of the ASVs were not recovered in our study. Firstly, DNA sequencing techniques usually do not differentiate between live and dead cells, which can lead to an overestimation of the presence of metabolically active cells in the ecosystem [81, 82]. In addition, among the active bacteria, we can found the so-called viable but non culturable (VBNC) bacteria, which are unable to form colonies in solid media [83]. As a result, it is possible that some of the ASVs we detected belong to these dead cells or the VBNC bacteria, which we would not be able to recover through isolation. On the other hand, the culturing conditions used were focused on the retrieval of heterotrophic bacteria commonly isolated under laboratory conditions from different oceanographic regions and depths in order to be able to compare between samples. Future isolation efforts using dilution-to-extinction [13, 84], microdroplets or cultivation chips [85], methods combining cell sorting with isotopic labeling [86], or the use of metagenomes and metatranscriptomes data to predict the metabolic requirements of certain bacterial groups and adapt culture media accordingly [87], may help us to bring to the laboratory some other abundant and biogeochemically key taxa of the free-living microbial communities that remain mostly uncultured. Nevertheless, the genome sequencing of these deep ocean cultured abundant taxa in combination with transcriptomic analyses under different experimental scenarios of temperature, pressure, or particles association would enhance our understanding of key taxa of the deep ocean.

## Material and methods

### Isolates culture collection database

Water samples from a total of 12 photic-layer stations and 11 deep-ocean stations (including four from the mesopelagic in oxygen minimum zone (OMZ) regions and seven from the bathypelagic) were collected from global oceanographic expeditions including the Malaspina Expedition [88] and the *Tara* Oceans (*Tara* Oceans 2009 and *Tara* Oceans Polar Circle 2013) [53]. Additionally, we used seawater samples collected in other cruises, such as ATP09 in the Arctic Ocean [89], MIFASOL in the NW Mediterranean, as well as from the Blanes Bay Microbial Observatory (BBMO, http://www.icm.csic.es/bio/projects/icmicrobis/bbmo), covering a wide latitudinal range, with different oceanographic regions. Seawater samples collected for isolation were prefiltered through 200 µm and 20 µm mesh in succession in order to keep free-living bacteria but also the prokaryotic community attached to particles <20 µm. Further details regarding sample collection in all these cruises have been previously described [47]. Geographical coordinates of the stations, sampled depth, *in situ* temperature, number of isolates sequenced, total prokaryote cell abundances, and colony forming units (cfu) per mL are listed in Supplementary Table S11. Prokaryote cell abundance was determined using flow cytometry (in a Becton Dickinson FACSCalibur) of SYBR Green I stained samples [90].

Our culturing strategy was focused on retrieving heterotrophic marine bacteria that could easily grow under laboratory conditions (nutrient rich media, standard oxygen concentrations and atmospheric pressure) (Supplementary Table S12). Therefore, we used nutrient rich media including Zobell Agar, Marine Agar 2216 and modified Marine Agar, where disodium phosphate was autoclaved separately from the rest of the media and added as a separate solution before solidification [47, 91]. We are aware that the use of the modified Marine Agar only from some stations could bias our results due to the possibility to isolate different strains. Accordingly, some of the analyses were also performed excluding the isolates obtained from this specific medium. The results generated (Supplementary Fig. S12) agreed whether we included those isolates or not, which allowed us to keep them for the remaining analyses. Further details regarding isolation of bacterial strains has been already described in Sanz-Sáez et al. (2020) [47].

A total of 2003 bacterial isolates (MARINHET_v2 culture collection) were randomly selected based on different colony morphology for DNA amplification and partial sequencing of their 16S rRNA gene (more details in ref. [47]). Similar number of isolates were selected from photic layers (1041; average: 70.6 isolates per station) and from the deep ocean (962; average: 67.6 isolates per station).

### Metabarcoding 16S rRNA datasets

We used metabarcoding 16S rRNA sequences obtained from several datasets and expeditions: Malaspina Surface (124 samples) [54], Malaspina Bathypelagic (41 samples, average depth: 3731 m ± 495; standard deviation) [46], *Tara* Oceans Surface (80 samples), *Tara* Oceans Mesopelagic (39 samples) (this study and Ibarbalz et al., 2019 [92]) (Fig. 1), and eight vertical profiles generated in the Malaspina Expedition that included five different size fractions for four depths corresponding to surface (3 m), the depth of the deep chlorophyll maximum (DCM, 48–150 m), mesopelagic (250–670 m), and bathypelagic waters (3105–4000 m) (Fig. 1A) [71]. These samples covered most tropical and temperate ocean regions but also some polar oceanic regions (*Tara* Oceans Polar Circle expedition Fig. 1A, B).

All samples were collected with 20 L Niskin bottles and were prefiltered through 200 µm and 20 µm mesh in succession. Volumes filtered and filters used for collecting prokaryotic DNA for analyses of the bacterial community using 16S amplicon Illumina TAGs are specified in Supplementary Table S13 for each cruise and type of sampling. Filters were then flash-frozen in liquid nitrogen and stored at −80 °C until DNA extraction.

### DNA extraction and sequencing for metabarcoding datasets

The samples for 16S metabarcoding sequencing were extracted with a phenol-chloroform protocol as previously described [46, 93, 94]. Prokaryotic barcodes for each of the datasets were generated by amplifying the V4 and V5 hypervariable regions of the 16S rRNA gene using primers 515F-Y (5’-GTGYCAGCMGCCGCGGTAA-3’) and 926 R (5′-CCGYCAATTYMTTTRAGTTT-3′) described in Parada et al. (2016) [55]. The Malaspina bathypelagic DNA samples originally from Salazar et al. (2016) [46] were re-sequenced again using 515F-Y-926R primers [55] to be comparable with the rest of the analyzed 16S metabarcoding datasets in this study. Sequencing for *Tara* Oceans, *Tara* Oceans Polar Circle and Malaspina Bathypelagic datasets was performed at Genoscope using an Illumina MiSeq platform (TAGs) with the 2 × 250 bp paired-end approach. The Malaspina Surface and Malaspina vertical-size fraction profiles datasets were sequenced at the Research and Testing Laboratory facility (https://rtlgenomics.com) also with Illumina MiSeq platform and the 2 × 250 bp paired-end approach.

### Metabarcoding sequence data processing

All metabarcoding 16S rRNA amplicons (16S TAGs) were processed *de novo* through the bioinformatic pipeline described in the GitHub repository (https://github.com/SushiLab/Amplicon_Recipes) regardless they were previously analyzed and published. Dereplication, definition of zero-radius OTUs or amplicon sequence variants (ASVs) were performed with USEARCH v.10.0.240 [95] using UNOISE3 algorithm. ASVs were taxonomically annotated against the SILVA database v.132 (2018) with the lowest common ancestor (LCA) approach. Further details can be found in Supplementary Material and Methods. Each ASV table was randomly sampled down to lowest sampling effort using the function *rrarefy.perm* with 1000 permutations from the R package *EcolUtils* [96]. A summary of the total number of reads per dataset, sample with the lowest number of reads and total ASVs before and after rarefication is described in Supplementary Tables S2 and S3.

### Comparison between 16S amplicon TAGs and cultured isolates

The primers used to obtain the 16S rRNA genes of the isolates were different from the ones used to obtain the 16S rRNA gene TAGs, although both amplified the V4 and V5 hypervariable region of the 16S rRNA gene. Therefore, comparisons between isolates and 16S TAGs were performed by selecting this common region (Supplementary Fig. S13).

All isolate sequences were compared to the ASVs at 100% similarity in order to have the strictest comparison possible. Comparisons were done by running global alignments using the *usearch_global* option from the USEARCH v10.0.240 [95]. The results were filtered by coverage of the alignment at 100% (i.e. all the ASVs sequences must align without any gaps in the sequence with the partial sequences of the isolates). We are aware that these comparisons sometimes result in more than one sequence hit per isolate. Nevertheless, all datasets contained similar proportions of isolates with more than one sequence match, and the % of reads recruited by these extra hits was minor (approximately 1.2% in each dataset), making comparisons between datasets possible (Supplementary Fig. S3).

### Data analysis

All data analyses were done with the R Statistical Software [97] using v.3.4.3 and the following packages: *vegan* [98], *ape* [99], *EcolUtils* [96], *stats* [97], *tidyverse* [100]. For the MARINHET_v2 culture collection accumulation curves and rarefaction curves were performed with OTUs (isolated OTUs) obtained after clustering of the 16S rRNA sequences at 100% similarity using the USEARCH v10.0.240 [95]. For metabarcoding 16S TAGs datasets, we calculated the mean abundance and relative abundance of ASVs across samples in order to rank the organisms detected. Moreover, we calculated the mean percentage of 16S TAGs reads (how much the isolates represent the bacterial community in terms of abundance?) and the ASVs (how much the isolates represent the bacterial community in terms of diversity or richness?) of the bacterial community that matched at 100% similarity with the 16S rRNA gene sequences of the strains isolated by traditional culture techniques. These percentages were calculated from the rarefied ASV-abundance tables. In order to test the significance of the differences between datasets we used non-parametric Kruskal–Wallis followed by post hoc pairwise Wilcox test. To assess significance, the statistical analyses were set to an alpha value of 0.05.

We used the 16S TAGs dataset from the Malaspina size fractionated vertical profiles to investigate whether our isolates were enriched in the free-living microbial fraction (0.2–0.8 μm), or in the particle-attached community, considering that this last category can be divided into four different size-fractions (0.8–3 μm, 3–5 μm, 5–20 μm and 20–200 μm). Hence, we also calculated the percentage of reads or 16S TAGs, and ASVs that matched at 100% similarity with our isolates. The differences between size fractions were also tested with the non-parametric Kruskal–Wallis test followed by the post hoc pairwise Wilcox test. Again, the significance was set at an alpha value of 0.05.

### Nucleotide accession numbers

The isolates sequences are deposited in GenBank under accession numbers MH731309 - MH732621 and MK658870-MK659428. Amplicon 16S rRNA TAGs from the different datasets used are available in the European Nucleotide Archive (ENA). Those from the Malaspina Surface dataset are available under accession number PRJEB25224, those from the Malaspina Bathypelagic dataset under PRJEB45011, those from Malaspina size fractions under PRJEB27154 and those from *Tara* Oceans and *Tara* Oceans Polar Circle under accession numbers PRJEB36282, PRJEB36283 and PRJEB36439.

### Supplementary information

Supplementary Information

Supplementary Tables

## Data Availability

All isolates data is available in GenBank under accession numbers MH731309 - MH732621 and MK658870-MK659428; and amplicon 16S TAGs in the European Nucleotide Archive (ENA) under accession numbers PRJEB25224, PRJEB45011, PRJEB27154, PRJEB36282, PRJEB36283 and PRJEB36439.
