# Supplementary material for: Top abundant deep ocean heterotrophic bacteria can be retrieved by cultivation
Source: ISME Commun. 2023 Sep 2;3:92. doi: 10.1038/s43705-023-00290-0 (PMC10475052; doi:10.1038/s43705-023-00290-0)
Supplement: Supplementary file 1 — Supplementary Information [file 43705_2023_290_MOESM1_ESM.pdf]

Supplementary Information for:

Top abundant deep ocean heterotrophic bacteria can be retrieved by cultivation

Isabel Sanz-Sáez*, Pablo Sánchez, Guillem Salazar, Shinichi Sunagawa, Colomban de Vargas, Chris Bowler, Matthew B. Sullivan, Patrick Wincker , Eric Karsenti, Carles Pedrós-Alió, Susana Agustí, Takashi Gojobori, Carlos M. Duarte, Josep M. Gasol, Olga Sánchez*, Silvia G. Acinas[1*]

Corresponding authors: Isabel Sanz-Sáez: isanz@icm.csic.es or isscgg@cid.csic.es; Olga Sánchez: olga.sanchez@uab.es; Silvia G. Acinas: sacinas@icm.csic.es

This PDF file includes:

SI Methodology

SI Results

SI Figures S1 to S13

SI Tables S1 to S13

SI References

Other Supplementary Material for this manuscript include the following:

Excel file with Supplementary Tables S1 and S5

SI Methodology

Metabarcoding sequence data processing

All metabarcoding 16S rRNA amplicons were processed *de novo* through the bioinformatic pipeline described in the GitHub repository (https://github.com/SushiLab/Amplicon_Recipes) regardless they were previously analyzed and published. Pair-end reads were merged at a minimum 90% of identity alignment, and those with ≤ 1 expected errors were selected (quality filtering). Primer matching was performed with CUTADAPT v.1.9.1. Dereplication, definition of zero-radius OTUs or amplicon sequence variants (ASVs) were performed with USEARCH v.10.0.240 [1] using UNOISE3 algorithm. ASVs were taxonomically annotated against the SILVA database v.132 (2018) with the lowest common ancestor (LCA) approach. This procedure was applied individually for the: (i) 41 Malaspina Bathypelagic samples, (ii) 124 Malaspina Surface samples, (iii) 119 *Tara* Oceans and *Tara* Oceans Polar Circle Surface and Mesopelagic samples, and (iv) 155 samples from the Malaspina vertical-size fractions profiles dataset. Hence, four different and new ASV-abundance tables were obtained after applying the above pipeline (Malaspina datasets were re-preprocessed from the original publications of the samples [2–4]). Non-prokaryotic ASVs (eukaryotes, chloroplasts and mitochondria) were removed, whereas singletons (ASVs appearing only once) were maintained. For some specific analyses, Cyanobacteria or Archaea reads were also removed since our isolation protocol was not adequate for culturing these microorganisms. Computing analyses were run at the MARBITS bioinformatics platform at the *Institut* de *Ciències del Mar* and at the Euler scientific compute cluster of the ETH Zürich University.

Extra analyses at 97% sequence similarity

The results presented in this study are based on the detection of amplicon sequence variants (ASVs) compared to 16S rRNA isolates sequences at 100% sequence similarity. However, during the analyses we also defined from metabarcoding 16S TAGs OTUs at 97% with the UPARSE algorithm using USEARCH v.10.0.240 [1]. OTUs at 97% identity and ASVs were taxonomically annotated against the SILVA database v.132 (2018) with the lowest common ancestor (LCA) approach. OTU-abundance tables were also defined for OTUs at 97% sequence similarity. OTUs at 97% sequence similarity were then compared to 16S rRNA isolates sequences at 97% sequence similarity. Some results about these comparisons can be found in **Supplementary Table S2 and S3**.

SI Results

ASVs vs 16S rRNA isolates sequences comparisons

We determined the mean percentage of ASVs (diversity) as well as the mean percentage of 16S TAGs (abundance) that were 100% identical to our MARINHET_v2 culture collection isolates. Besides the global comparisons (comparing each 16S TAGs dataset from *Tara* Oceans and Malaspina Expedition with all the isolate sequences) two other types of comparisons were done: (i) comparing separately the photic and aphotic 16S TAGs datasets with only photic or aphotic zone isolate sequences, and (ii) comparing the 16S TAGs and the rRNA gene sequences of the isolates retrieved from exactly the same stations. A summary of the results of these three levels of comparisons, obtained from the rarefied ASVs-abundance table, is shown in **Supplementary Table S3 and Figure S7**.

When the comparisons of the datasets were performed separately for the photic and aphotic zone isolates, the highest mean % of ASVs equal to isolates was found in Malaspina Surface (2.7% photic, 4.1% aphotic) and the lowest percentages were found in *Tara* Oceans Mesopelagic (0.9% photic, 1.1% aphotic) (**Supplementary Table S3).**

SI Figures

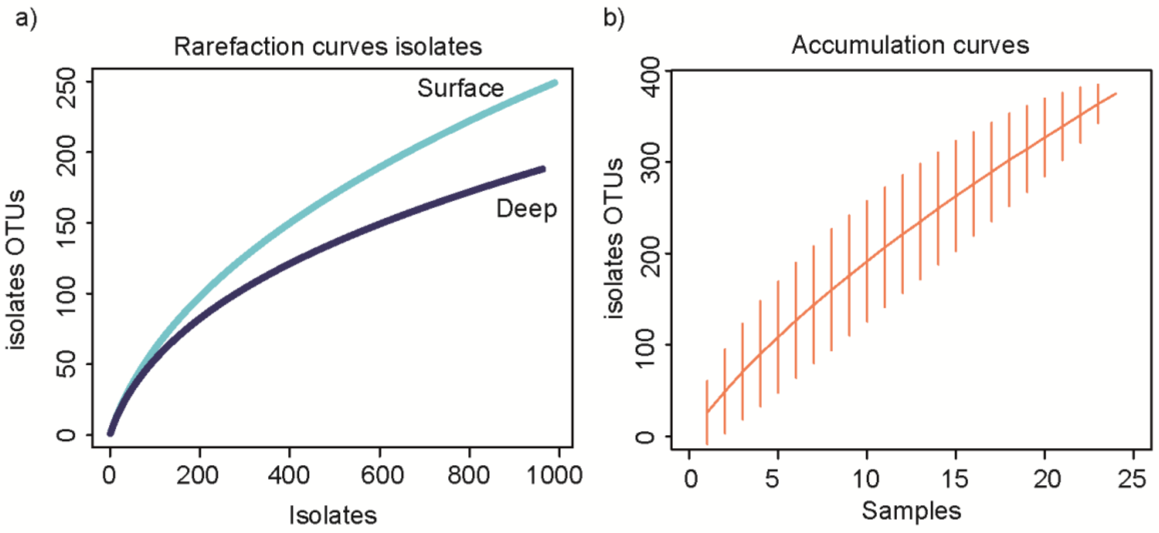

Figure S1. Description of the isolation effort per layer. (A) Rarefaction curves of the photic (light-blue) versus aphotic (dark-blue) isolates. **(B)** Accumulation curves of the total isolates dataset. Plots are generated from the non-rarefied isolates OTUs (clustering of the isolates 16S rRNA sequences at 100% sequence similarity). See Materials and Methods for an explanation of how OTUs isolates were obtained.

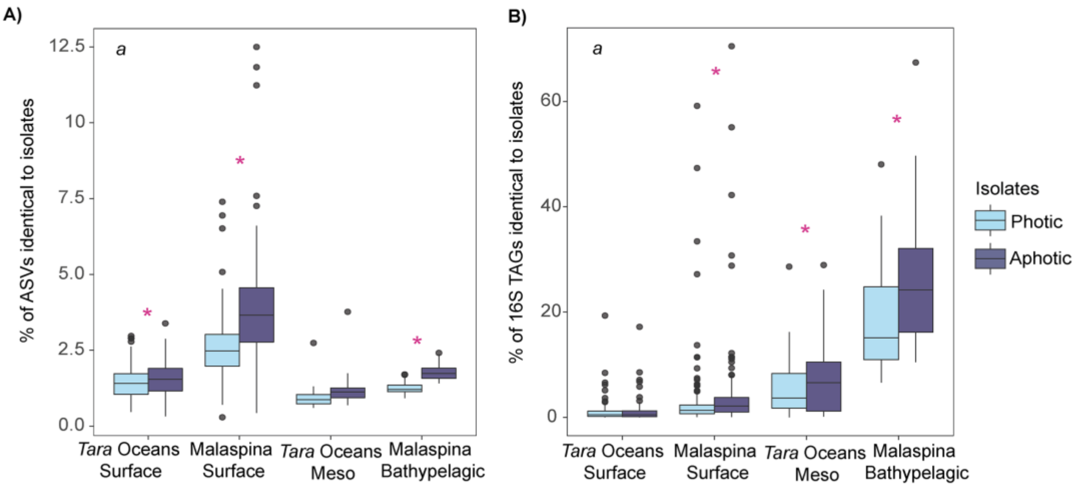

Figure S2. Summary of the percentages of ASVs or 16S TAGs identical to isolates in the datasets analyzed separating photic and aphotic zone isolates. (A) Proportion of isolates that are 100% similar to the ASVs comparing photic and aphotic zone isolates separately. **(B)** Proportion of 16S TAGs reads matching isolates at 100% similarity when comparing photic and aphotic zone isolates separately. Significative differences between datasets (P-value < 0.01) are indicated by an italic *a* in the top left corner, while significative differences (P-value < 0.01) within a dataset are indicated by pink asterisks.

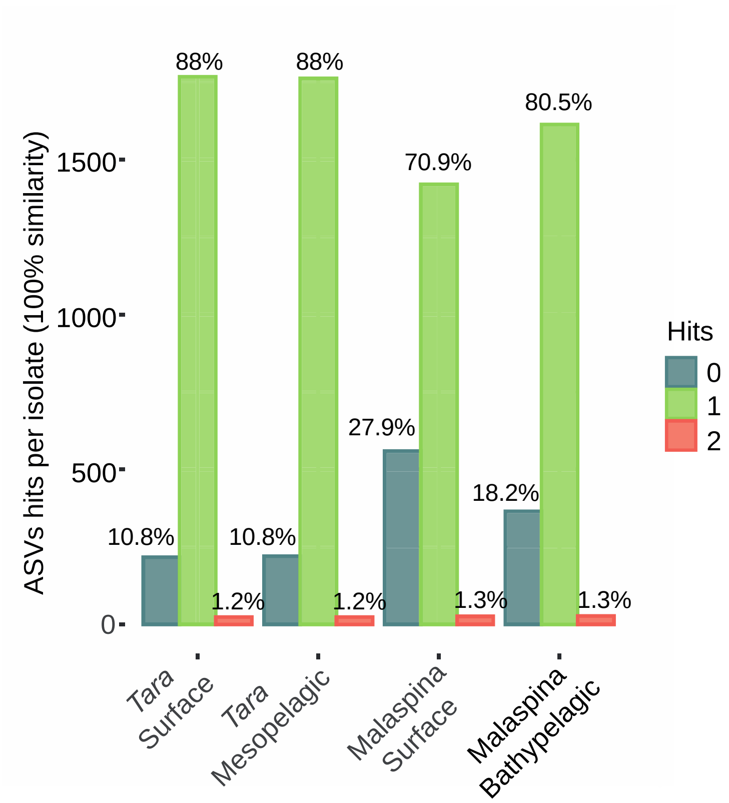

Figure S3. Proportion of isolates identical to ASVs. Number of isolates presenting no hits to any ASV (0 hits, dark-green), just hits to one single ASV (1 hit , pale green) or hits to 2 different ASVs (2 hits, orange). Comparisons done at 100 % sequence similarity using *usearch_global*. Percentages in each category from the total isolates are indicated above each bar.

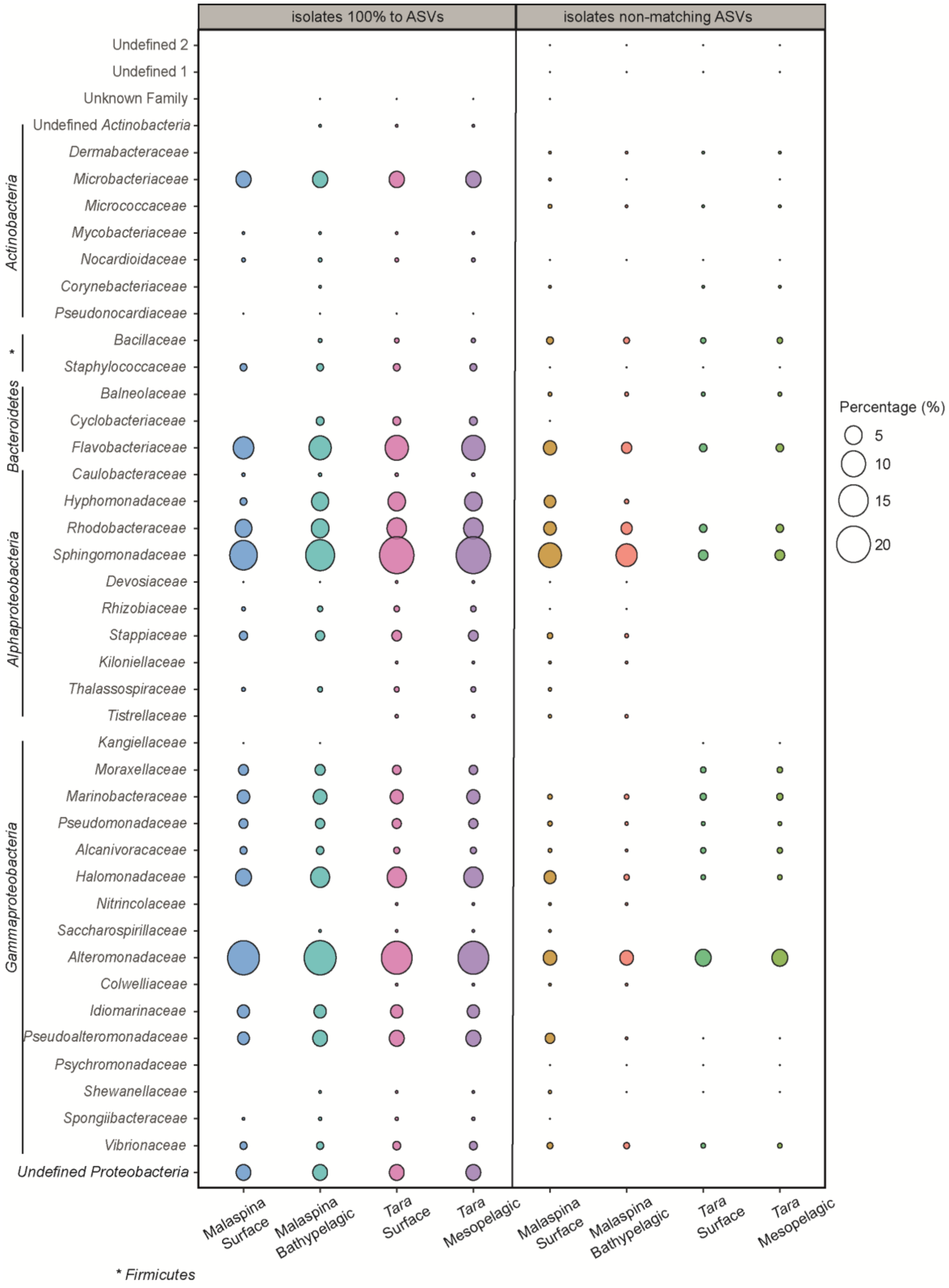

Figure S4. Taxonomic differences at the family level between those isolates that were 100% identical to ASVs in the Malaspina Surface, Malaspina Bathypelagic, *Tara* Oceans Surface and *Tara* Oceans Mesopelagic datasets. The first four columns represent those isolates 100% identical to ASVs, and the last four columns represent

those isolates that did not match any ASV. The size of the dots indicates the percentage of isolates in each family from the total isolates of the MARINHET_v2 collection.

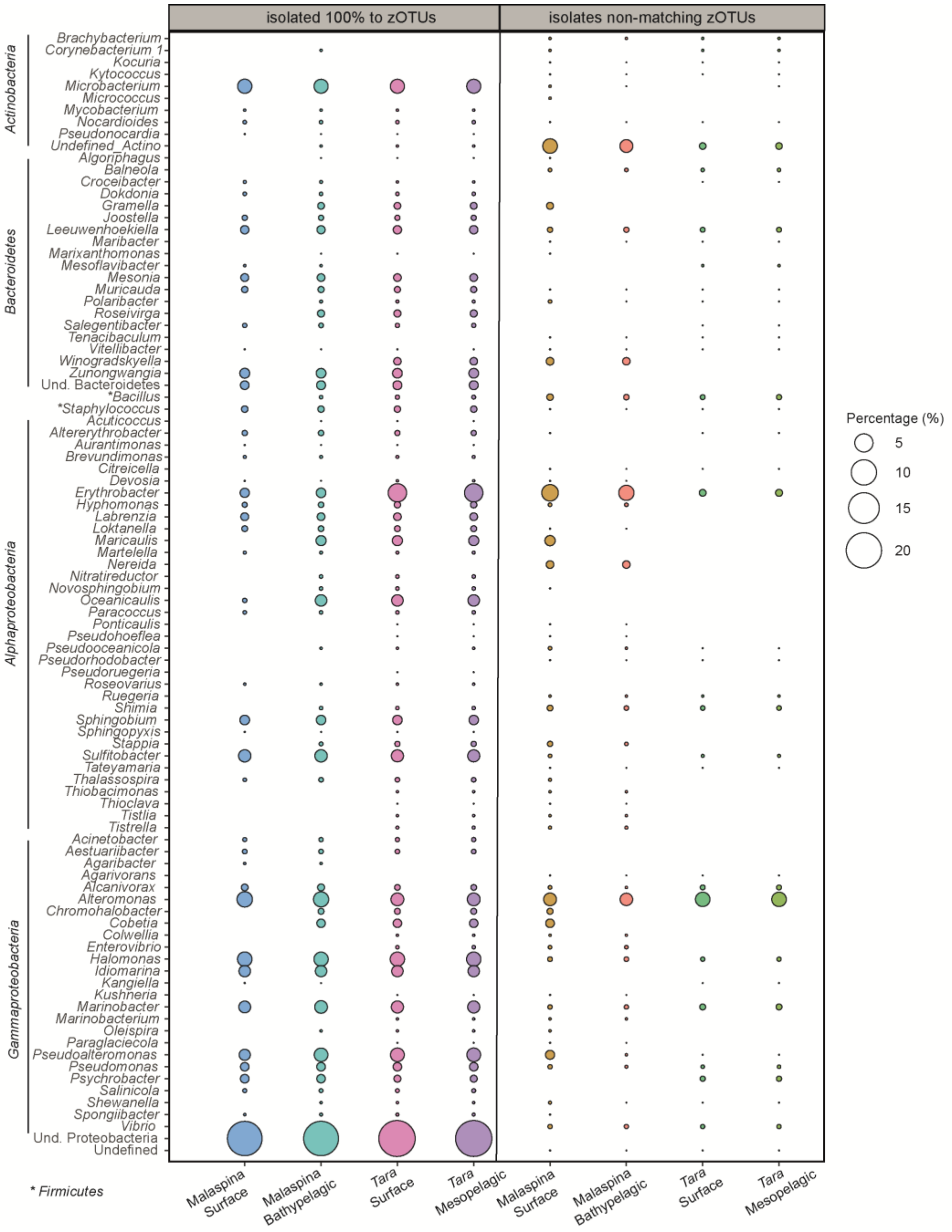

Figure S5. Taxonomic differences at the genus level between those isolates that were 100% identical to ASVs in the Malaspina Surface, Malaspina Bathypelagic, *Tara* Surface and *Tara* Mesopelagic datasets. The first four columns represent those isolates 100% identical to ASVs, and the last four columns represent those isolates that did not match with any of the ASVs. The size of the dots indicates the percentage of isolates in each family from the total isolates of the MARINHET_v2 collection.

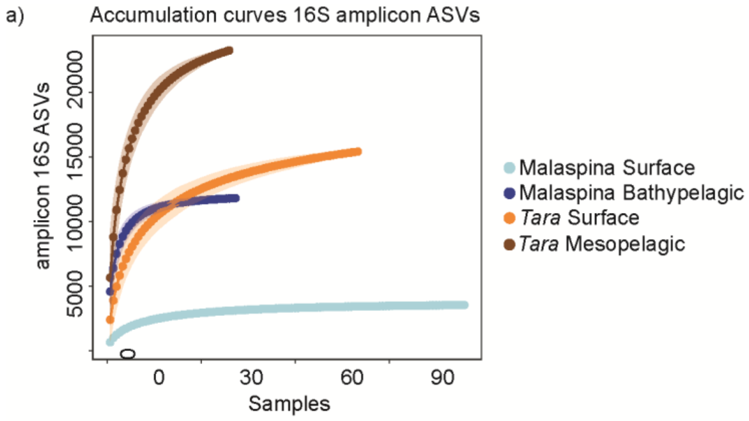

Figure S6. Accumulation curves of the metabarcoding 16S TAGs datasets including Malaspina Surface, Malaspina Bathypelagic, *Tara* Surface and *Tara* Mesopelagic.

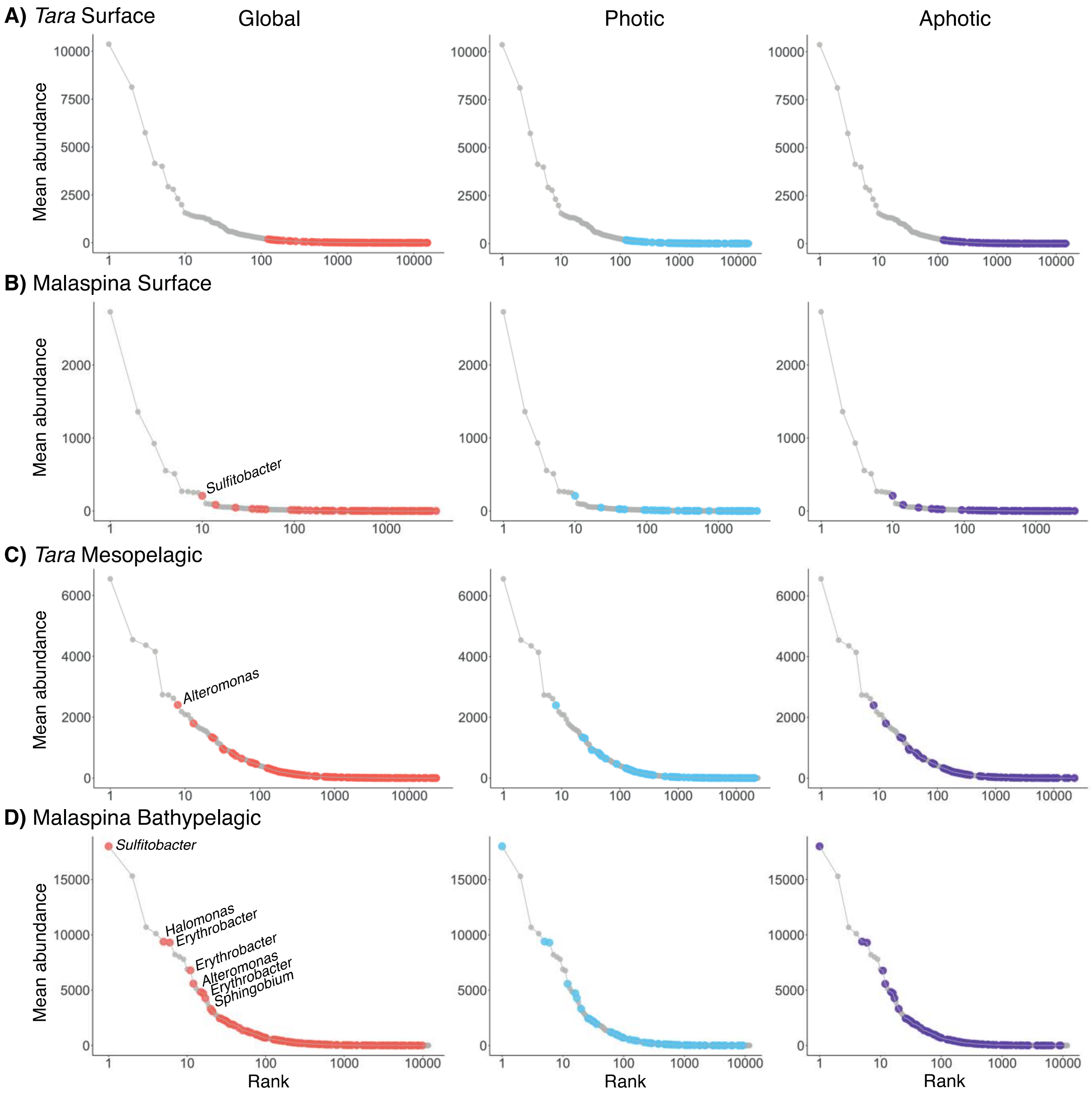

Figure S7. Rank plots showing the ASVs recruited by photic and aphotic ocean isolates by each different dataset. (A) *Tara* Surface. **(B)** Malaspina Surface. **(C)** *Tara* Mesopelagic. **(D)** Malaspina Bathypelagic. Colored dots indicate which ASVs are 100% identical to at least one isolate in the comparisons made with all photic and aphotic depth isolates (global), and separately with the photic and aphotic datasets: grey, ASVs that did not match any isolate; orange, blue or purple, ASVs 100% identical to isolates. Taxonomic affiliation is indicated for the abundant (>1 % abundance) ASVs that are identical to isolates in the global rank plot.

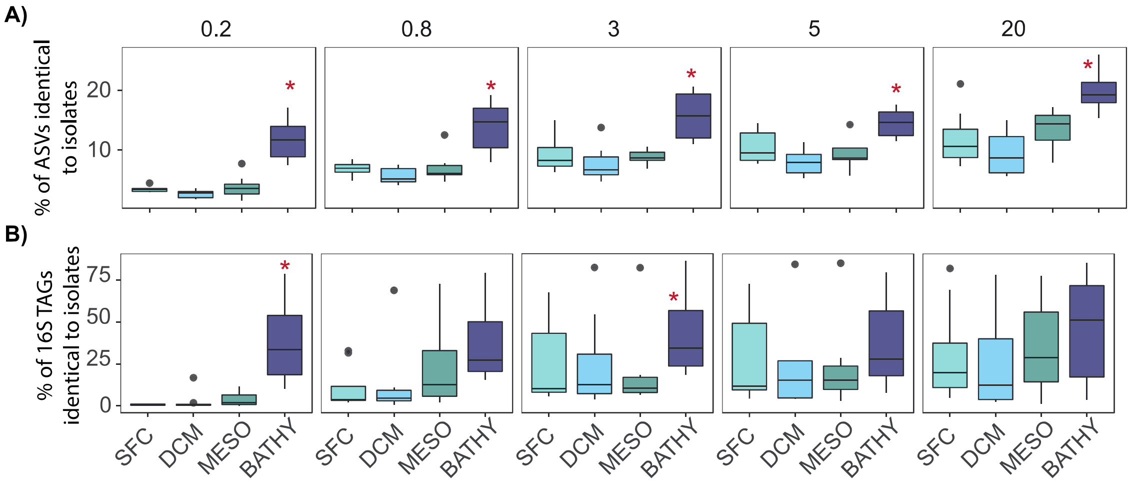

Figure S8. **Boxplots comparing different size fractions in the Malaspina profiles showing: (A)** percentages of ASVs that were 100% identical to at least one isolate per size fractions and depth, and **(B)** percentages of 16S TAGs (reads) that were 100% identical to at least one isolate per size fractions and depth. SFC: surface, DCM: deep chlorophyll maximum, MESO: mesopelagic, and BATHY: bathypelagic. 0.2: free-living bacteria, 0.8: bacteria attached to small particles, and 3.0-20: bacteria attached to larger particles. Units of the size fractions are in µm. Significative differences between layers (Kruskal-Wallis, P-value from 1.1×10^{-8} to 4.7×10^{-12}) are indicated by red asterisks.

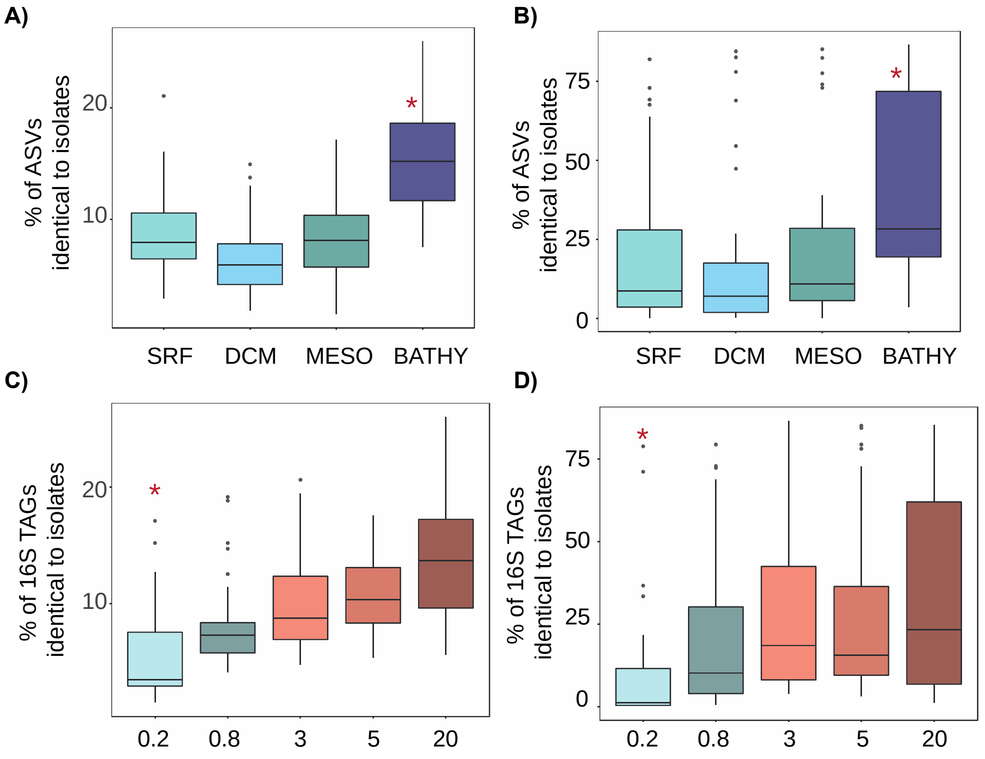

Figure S9. Boxplots comparing different size fractions in the Malaspina profiles showing: (A,B) percent of ASVs and 16S TAGs, respectively, that were 100% identical to at least one isolate per depth, and **(C,D)** percent of ASVs and 16S TAGs, respectively, that were 100% identical to at least one isolate per size fraction. SFC: surface, DCM: deep chlorophyll maximum, MESO: mesopelagic, and BATHY: bathypelagic. 0.2: free-living bacteria, 0.8: bacteria attached to small particles, and 3-20: bacteria attached to larger particles. Units of the size fractions are in µm. Significative differences between layers or size fractions (Kruskal-Wallis, P-value <0.05) are indicated by red asterisks.

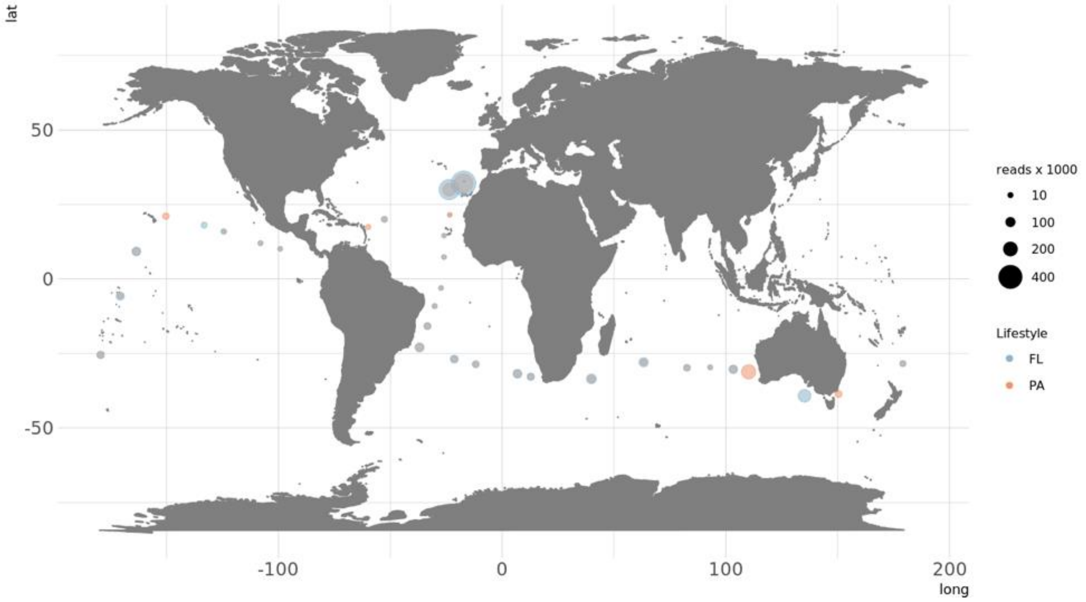

Figure S10. Biogeography map showing the abundance (reads x 1000) of the MAG0295 from Deep Malaspina MAGs dataset associated to *Sulfitobacter pontiacus* based on GTDB.

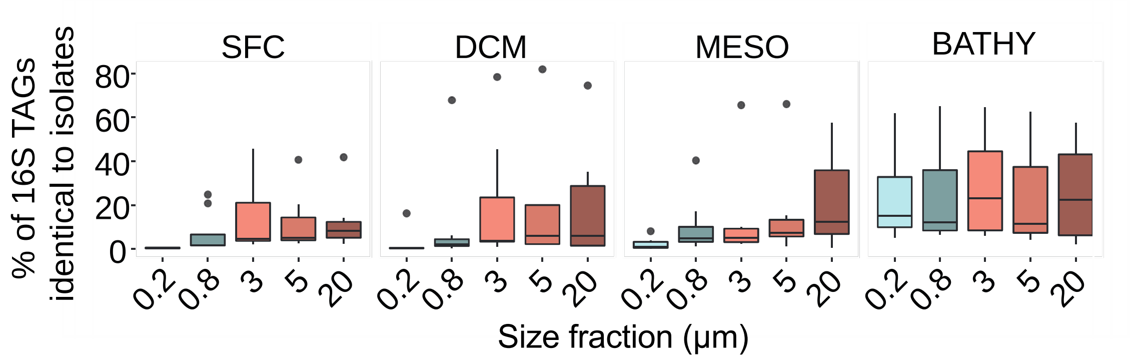

Figure S11. Distribution of bathypelagic abundant ASVs (>1 % of the reads) in all layers and size fractions. The values represent the mean abundance of reads across layers and fractions. SFC: surface, DCM: deep chlorophyll maxima, MESO: mesopelagic, and BATHY: bathypelagic. 0.2: free-living bacteria, 0.8: bacteria attached to small particles, and 3-20: bacteria attached to large particles.

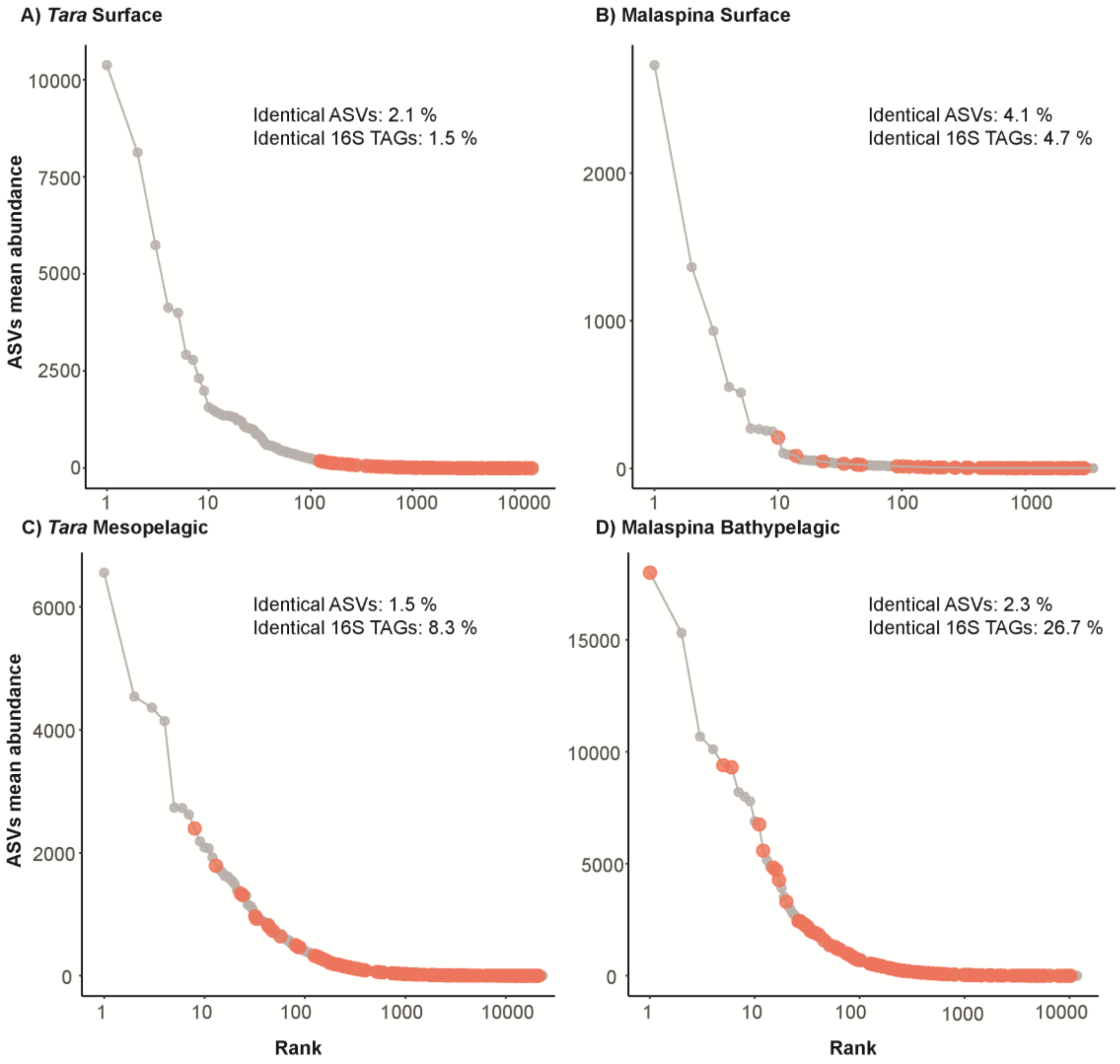

Figure S12. Rank plots showing the identified isolates that matched at 100% identity to 16S TAGs in different datasets excluding those isolates retrieved with a different set of incubation conditions (modified Marine Agar). (A) *Tara* Oceans Surface **(B)** Malaspina Surface **(C)** *Tara* Oceans Mesopelagic **(D)** Malaspina Bathypelagic. Colored dots indicate ASVs matched by isolates: grey, isolates that did not matched any ASVs; orange, isolates with 100% identity to ASVs.

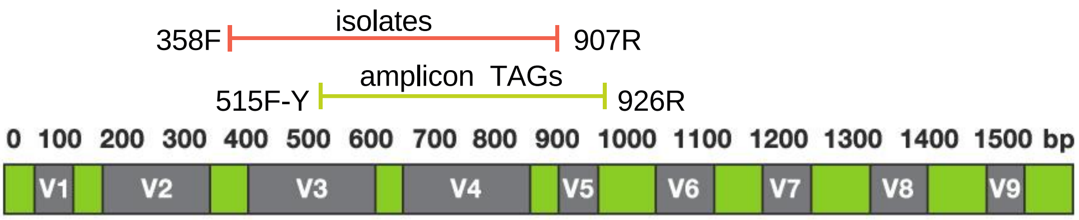

Figure S13. Graphic representation of the 16S rRNA gene and the regions covered by isolates (orange) and amplicon 16S TAGs (yellow). Forward and reverse primers used for 16S rRNA amplifications in isolates and amplicon TAGs are indicated at the left and right sides of the lines.

SI Tables

Table S1. Summary of the Genera retrieved in the MARINHET_v2 culture collection. Number of isolates retrieved and stations were strains were recovered is indicated. *** file in Excel document**

Table S2. Summary of the total reads (16S TAGs), total OTUs (97%) and ASVs (100%), lowest number of reads and reads after rarification for each *Tara* Oceans and Malaspina Expedition amplicon 16S TAGs datasets. OTUs at 97% sequence similarity were obtained using the UPARSE algorithm of USEARCH v.10.0.240 [1] just for comparative purposes.

Dataset	UPARSE 97%					UNOISE 100%				
	Total reads	Total OTUs	Lowest number of reads	Reads after rarification	OTUs after Rarification	Total reads	Total ASVs	Lowest number of reads	Reads after rarification	zOTUs after Rarification
Malaspina Surface	6.16E+06	11685	12049	1373586	4975	6.34E+06	3810	12115	1381110	3528
Malaspina Bathypelagic	3.96E+07	26311	431835	17705235	19591	3.98E+07	11963	434069	17796829	11820
Tara Surface	3.46E+07	82671	140076	11206080	37064	3.64E+07	17270	160849	12867920	15426
Tara Mesopelagic	1.84E+07	79691	230364	8984196	48476	1.90E+07	24435	236694	9231066	23274

Table S3. Proportions of ASVs 100 % similarity and OTUs at 97 % identical to isolates for the three levels of comparisons: global, photic and aphotic zones, and per stations used for isolation.

Dataset	Comparison		OTUs identical to isolates (%)		16S TAGs identical to isolates (%)	
			OTUs 97%	*ASVs 100%*	*OTUs 97%*	*ASVs 100%*
**Tara Surface**	All isolates		3.8	2.3	4.8	1.6
	Photic isolates		2.6	1.4	3.4	1.3
	Aphotic isolates		2.4	1.5	2.7	1.3
	Individual Surface Samples	ST39	0.3	0.2	1.7	1.4
		ST67	0.3	0.3	0.2	0.07
		ST72	0.6	0.4	1.8	1.2
		ST76	0.3	0.4	2.1	1.9
		ST151	0.8	0.4	1.7	0.9
		ST163	0.6	0.2	0.2	0.08
		ST84	0.5	0.3	0.08	0.07
		ST85	2.1	1.2	0.09	0.03
**Tara Mesopelagic**	All isolates		2.6	1.7	9.8	8.5
	Photic isolates		1.5	0.9	7.5	5.6
	Aphotic isolates		1.6	1.1	8.8	7.8
	Individual Mesopelagic Samples	ST102	0.2	0.2	11.2	9.2
		ST111	0.6	0.3	8.2	6
		ST138	0.7	0.4	9.5	6.1
**Malaspina Surface**	All isolates		8.2	4.5	6.2	4.8
	Photic isolates		7	2.7	5.6	3.3
	Aphotic isolates		6.6	4.1	5.2	4.5
**Malaspina Bathypelagic**	All isolates		3.1	2.4	35.7	27.9
	Photic isolates		2	1.2	29.1	18.4
	Aphotic isolates		2.1	1.8	32.4	26
	Individual Bathypelagic Samples	ST10	0.2	0.1	14.1	10.4
		ST17	0.6	0.3	26.3	21.6
		ST23	1.2	0.5	32.2	28.9
		ST32	0.9	0.3	29.3	18.2
		ST43	0.3	0.2	19.9	16.4

Table S4. Proportions of isolates 100 % identical to ASVs per dataset, and proportions of those that did not match with any ASV.

	**Malaspina Surface**	**Malaspina Bathypelagic**	***Tara* Surface**	***Tara* Mesopelagic**
Isolates 100% identical to ASVs (%)	1445 (72.1%)	1639 (81.8%)	1787 (89.2%)	1784 (89%)
Isolates with 1 ASV hit (%)	1420 (70.9%)	1613 (80.5%)	1763 (87.9%)	1762 (87.95)
Isolates with 2 ASVs hit (%)	26 (1.3%)	27 (1.3%)	24 (1.2%)	24 (1.2%)
Non-matching isolates (%)	559 (27.9%)	365 (18.2%)	217 (10.8%)	220 (10.9%)

Table S5. Taxonomic classification of the different ASVs that were 100% identical to isolates found in *Tara* Oceans and Malaspina Expedition amplicon 16S TAGs datasets.
*** file in Excel document**

Table S6. Proportion of 16S TAGs (reads) identical to isolates at 100 % similarity in the Malaspina size fraction dataset per layer and size fraction. SFC: surface, DCM: deep chlorophyll maximum, MESO: mesopelagic; BATHY: bathypelagic; sd, standard deviation; IQR, interquartile range. Mean and median are not a percentage (i.e. 0.1 will represent 10%).

**Proportion of 16s TAGs identical to isolates per layer and size fraction**						
DEPTH	FILTER	stations_cou	mean	sd	median	IQR
SFC	0.2	8	0.0	0.0	0.0	0.0
SFC	0.8	8	0.1	0.1	0.0	0.1
SFC	3	8	0.3	0.3	0.2	0.4
SFC	5	8	0.3	0.3	0.2	0.3
SFC	20	8	0.3	0.3	0.2	0.3
DCM	0.2	8	0.0	0.1	0.0	0.0
DCM	0.8	8	0.1	0.2	0.1	0.0
DCM	3	8	0.3	0.3	0.1	0.2
DCM	5	8	0.2	0.3	0.2	0.2
DCM	20	8	0.2	0.3	0.1	0.2
MESO	0.2	8	0.0	0.0	0.0	0.1
MESO	0.8	8	0.2	0.2	0.1	0.3
MESO	3	8	0.2	0.3	0.2	0.2
MESO	5	8	0.2	0.3	0.2	0.1
MESO	20	8	0.3	0.3	0.2	0.4
BATHY	0.2	7	0.4	0.3	0.3	0.4
BATHY	0.8	7	0.4	0.3	0.3	0.3
BATHY	3	7	0.4	0.3	0.3	0.3
BATHY	5	7	0.4	0.3	0.3	0.4
BATHY	20	7	0.5	0.3	0.5	0.5

Table S7. Proportion of ASVs identical to isolates at 100 % similarity in the Malaspina size fraction dataset per layer and size fraction. SFC: surface, DCM: deep chlorophyll maximum, MESO: mesopelagic; BATHY: bathypelagic; sd, standard deviation; IQR, interquartile range. Mean and median are not a percentage (i.e. 0.1 will represent 10%).

**Proportion of ASVs identical to isolates per layer and size fraction**						
DEPTH	FILTER	count	mean	sd	median	IQR
SFC	0.2	8	0.0	0.0	0.0	0.0
SFC	0.8	8	0.1	0.0	0.1	0.0
SFC	3	8	0.1	0.0	0.1	0.0
SFC	5	8	0.1	0.0	0.1	0.0
SFC	20	8	0.1	0.0	0.1	0.0
DCM	0.2	8	0.0	0.0	0.0	0.0
DCM	0.8	8	0.1	0.0	0.1	0.0
DCM	3	8	0.1	0.0	0.1	0.0
DCM	5	8	0.1	0.0	0.1	0.0
DCM	20	8	0.1	0.0	0.1	0.0
MESO	0.2	8	0.0	0.0	0.0	0.0
MESO	0.8	8	0.1	0.0	0.1	0.0
MESO	3	8	0.1	0.0	0.1	0.0
MESO	5	8	0.1	0.0	0.1	0.0
MESO	20	8	0.1	0.0	0.1	0.0
BATHY	0.2	7	0.1	0.0	0.1	0.1
BATHY	0.8	7	0.2	0.0	0.2	0.1
BATHY	3	7	0.2	0.0	0.2	0.1
BATHY	5	7	0.2	0.0	0.1	0.0
BATHY	20	7	0.2	0.0	0.2	0.0

Table S8. Summary of the proportions of ASVs and 16S TAGs identical (100% similarity) to isolates after removing *Cyanobacteria* (A) or *Archaea* (B) from the ASV-abundance tables in photic and aphotic zone *Tara* Oceans and Malaspina Expedition datasets.

A. ASVs identical to isolates without cyanos			
**Dataset**	**Comparison**	**% ASVs**	**% 16S TAGs**
Tara Surface	All isolates	2.9	2.9
Tara Meso	All isolates	2	10.9
Malaspina Surface	All isolates	6.1	10.1
Malaspina Bathypelagic	All isolates	2.7	29.1

B. ASVs identical to isolates without Archaea			
**Dataset**	**Comparison**	**% ASVs**	**% 16S TAGs**
Tara Surface	All isolates	2.23	1.61
Tara Meso	All isolates	1.9	11.63
Malaspian Surface	All isolates	4.64	4.96
Malaspina Bathypelagic	All isolates	2.60	31.43

Table S9. Summary of the rRNA average copy number for each of the genera identified in the MARINHET_v2 culture collection. Values extracted from: https://rrndb.umms.med.umich.edu/.

Phyllum	Genera	Number of rRNA operon copies	
		mean	sd
Actinobacteria	*Corynebacterium*	4.3	0.8
	Mycobacterium	1.1	0
	Microbacterium	2	0.4
	Nocardioides	2.4	0.8
	Pseudonocardia	3.7	0.8
	Undefined	NA	
Bacteroidetes	*Algoriphagus*	3	0
	Roseivirga	1	0
	Croceibacter	2	0
	Dokdonia	3	0
	Gramella	3	0
	Joostella	NA	
	Leeuwenhoekiella	3	0
	Marixanthomonas	2	0
	Mesoflavibacter	2	0
	Mesonia	NA	
	Muricauda	2	0
	Polaribacter	3.9	
	Salegentibacter	3	0
	Vitellibacter	2	0
	Winogradskyella	2.6	1.3
	Zunongwangia	3	0
	Flavobacteriaceae	4.6	2.9
Firmicutes	*Bacillus*	8.7	3.3
	Staphylococcus	5.7	0.7
Alphaproteobacteria	*Acuticoccus*	2	0
	Brevundimonas	2	0.4
	Hyphomonas	1	0
	Maricaulis	2	0
	Oceanicaulis	1.3	0.4
	Ponticaulis	1.2	0.4
	Devosia	2.4	1.4
	Aurantimonas	2	0
	Martelella	2.5	
	Nitratireductor	1.7	0.5
	Pseudohoeflea	1.9	0.5
	Labrenzia	2.9	0.3
	Stappia	2.8	0.4
	Loktanella	2.7	1.2
	Paracoccus	2.7	0.5
	Pseudooceanicola	2	0
	Pseudoruegeria	2.7	1.2
	Roseovarius	1.2	0.4
	Shimia	2.7	1.2
	Sulfitobacter	2.5	1.2
	Thiobacimonas	NA	
	Thioclava	3	0
	Thalassospira	4	1.1
	Tistilla	NA	
	Tistrella	4	0
	Altererythrobacter	1.3	0.4
	Erythrobacter	1.7	1
	Novosphingobium	2.8	1.3
	Sphingobium	2.9	1
	Sphingopyxis	1.1	0.2
	Sphingimonadaceae	2.1	1
	Rhodobacteraceae	2.8	1.1
Gammaproteobacteria	*Aestuariibacter*	6.2	2.5
	Agaribacter	6.2	2.5
	Alteromonas	7.1	2.2
	Paraglaciecola	5.3	0.4
	Colwellia	6.7	1.2
	Idiomarina	4	0
	Marinobacter	3.3	0.9
	Pseudoalteromonas	8.7	1.1
	Shewanella	8.8	1.6
	Spongiibacter	2.3	0.4
	Alcanivorax	2.4	0.5
	Chromohalobacter	5	0
	Cobetia	6.9	0.3
	Halomonas	5.3	1.1
	Kushneria	4	0
	Salinicola	5	0
	Marinobacterium	4.8	1.1
	Kangiella	2	0
	Oleispira	5.6	2
	Acinetobacter	6.2	0.5
	Psychrobacter	4.4	0.9
	Pseudomonas	4.9	1.2
	Alteromonadaceae	6.2	2.5
	Vibrionaceae	10	3.3
	Undefined	NA	
AVERAGE rRNA copy number		3.51578947	--

Table S10. Mean abundance of the most abundant ASVs (>1% reads) in the bathypelagic (BATHY) samples extracted from the Malaspina size fraction dataset and their respective mean abundances in the surface (SFC), deep chlorophyll maxima (DCM) and mesopelagic (MESO) samples per size fraction.

**Proportion of bathypelagic abundant ASVs identical to isolates per layer and size fraction**				
**FILTER**	**DEPTH**	**count**	**mean abundance**	**% mean abundance**
0.2	BATHY	7	0.24	7.62
0.8	BATHY	7	0.25	7.84
3	BATHY	7	0.28	9.10
5	BATHY	7	0.24	7.63
20	BATHY	7	0.26	8.24
0.2	DCM	8	0.02	0.73
0.8	DCM	7	0.12	3.71
3	DCM	8	0.19	6.17
5	DCM	5	0.22	7.14
20	DCM	6	0.21	6.59
0.2	MESO	8	0.02	0.69
0.8	MESO	8	0.10	3.25
3	MESO	6	0.15	4.85
5	MESO	8	0.15	4.75
20	MESO	7	0.22	7.10
0.2	SFC	6	0.00	0.10
0.8	SFC	8	0.07	2.19
3	SFC	7	0.14	4.63
5	SFC	7	0.12	3.86
20	SFC	8	0.12	3.78

Table S11. Characteristics of the different samples from where isolates of marine heterotrophic bacteria were obtained. 'Non-redundant isolates' are the isolates remaining after duplicates (100% identical sequences in their partial 16S rRNA gene were removed).

Expedition	Station	Ocean	Latitude	Longitude	Depth (m)	*In situ* temperature (ºC)	Nº of sequenced isolates	Nº of non-redundant isolates	Cfu/ml	Cells/ml
Tara Oceans	ST 39	Indian Ocean	19º 2.24' N	64º 29.24' E	5.5	26.2	109	25	2.83E+03	9.70E+05
	ST 39	Indian Ocean	18º 35.2' N	66º 28.22' E	25	26.8	243	53	NA	9.50E+05
	ST 39	Indian Ocean	18º 43.12' N	66º 21.3' E	268.2	15.6	88	18	NA	9.70E+05
	ST 67	South Atlantic	32º 17.31' S	17º 12.22' E	5	12.8	115	49	2.24E+03	1.90E+06
	ST 72	South Atlantic	8º 46.44' S	17º 54.36' W	5	25	71	33	1.01E+04	6.90E+05
	ST 76	South Atlantic	20º 56.7' S	35º 10.49' W	5	23.3	89	27	1.06E+03	6.10E+05
	ST 84	Southern Ocean	60º 13.4' S	60º 38.51' W	5.9	1.8	10	8	1.28E+02	2.65E+05
	ST 85	Southern Ocean	62º 2.19' S	49º 31.44' W	5.9	0.7	126	30	4.28E+02	4.11E+05
	ST 85	Southern Ocean	62º 2.19' S	49º 31.44' W	87.4	-0.8	13	10	7.00E+01	2.02E+05
	ST 102	Pacific Ocean	5º 16.12' S	85º 13.12' O	475.6	9.2	97	15	2.35E+03	1.60E+05
	ST 111	Pacific Ocean	16º 57.36' S	100º 39.36'O	347.1	10.9	98	35	1.66E+03	6.50E+04
	ST 138	Pacific Ocean	6º 22.12' N	103º 4.12' O	444.9	8.2	91	34	1.12E+03	1.30E+05
	ST 151	North Atlantic	36º 10.17' N	29º 1.23' W	5	17.3	76	33	2.28E+03	4.40E+05
	ST 163	Arctic Ocean	76º 10.57' N	1º 23.31' E	5	1.9	18	7	8.17E+01	1.80E+05
	ST 175	Arctic Ocean	79º 13.24' N	66º 20.37' E	5	1.4	3	3	2.50E+01	4.21E+05
	ST 201	Arctic Ocean	74º 17.23º N	85º 48' W	5	-1.3	1	1	NA	5.88E+02
ATP 09	AR_1	Arctic Ocean	78º 20.00' N	15º 00.00' E	2	6.2	13	9	NA	NA
	AR_2	Arctic Ocean	76º 28.65' N	28º 00.62' E	25	-1.2	20	9	NA	NA
Malaspina	ST 10	North Atlantic	21º 33.36' N	23º26' W	4002	2.04	20	9	5.00E+01	3.30E+04
	ST 17	South Atlantic	3º 1.48' S	27º 19.48' W	4002	1.74	93	24	7.73E+02	3.00E+04
	ST 23	South Atlantic	15º 49.48' S	33º 24.36' W	4003	1.45	94	39	4.38E+02	1.20E+04
	ST 32	South Atlantic	26º 56.8' S	21º 24' W	3200	2.5	62	20	1.90E+02	1.80E+04
	ST 33	South Atlantic	27º 33.2' S	18º 5.4' W	3904	1.7	28	13	3.70E+01	1.50E+04
	ST 43	South Atlantic	32º 48.8' S	12º 46.2' E	4000	1.2	46	19	3.00E+01	3.80E+04
MIFASOL	ST 8	NW Mediterranean	40º 38.41' N	2º 50' E	2000	13.2	245	62	5.54E+02	2.20E+04
BBMO	BBMO	NW Mediterranean	41º 40' N	2º 48' E	5	17.71	134	62	NA	6.70E+05

Table S12. Culture media and incubation conditions used for each sample. Positive signs indicate which media were used. RT: room temperature.

Cruise	Depth and station	Tº	Zobell agar	Marine agar 2216	modified marine agar 2216
Tara Oceans	5 m (stations 67, 72, 76, 151)	RT	-	+	-
	5 m (stations 84, 85, 163, 175, 201)	RT/4ºC	-	-	+
	25 m (station 85)	RT/4ºC	-	-	+
	25 m (station 39)	RT	+	-	-
	OMZ (stations 39, 102, 111, 138)	RT	+	-	-
ATP 09	5 m	4ºC	+	-	-
	25 m	4ºC	+	-	-
Malaspina	4000 m (stations 10, 17, 23)	RT	-	+	-
	4000 m (stations 32, 33, 43)	RT/4ºC	+	+	+
Mifasol	2000 m	RT/12ºC	+	+	+
BBMO	5 m	RT	+	+	+

Table S13. Volumes and filters used for collecting prokaryotic DNA. Fractions analyzed in each dataset it is also indicated.

	**Malaspina Surface (3m)**	**Malaspina Bathypelagic**	**Malaspina vertical-profiles***	***Tara* Oceans and *Tara* Oceans Polar Circle**
Volume (L)	6-15	120	10	100
Prefilters (µm)	200/20	200/20	200/20	200/20
Filters (µm)	3.0/0.2	0.8/0.2	5.0/3.0/0.8/0.2	3/0.2 or 1.6/0.2
Fractions analyzed	0.2 – 3	0.2 – 0.8<br>0.8 – 20	0.2 – 0.8<br>0.8 – 3.0<br>3.0 – 5.0<br>5.0 – 20<br>20 – 200	0.2 – 1.6<br>Or<br>0.2 – 3.0

* Surface, deep chlorophyll maximum (DCM), mesopelagic, and bathypelagic samples

SI References

1. Edgar RC. Search and clustering orders of magnitude faster than BLAST. *Bioinformatics* 2010; **26**: 2460–2461.

2. Salazar G, Cornejo-Castillo FM, Benítez-Barrios V, Fraile-Nuez E, Álvarez-Salgado XA, Duarte CM, et al. Global diversity and biogeography of deep-sea pelagic prokaryotes. *ISME J* 2016; **10**: 596–608.

3. Ruiz-González C, Logares R, Sebastián M, Mestre M, Rodríguez-Martínez R, Galí M, et al. Higher contribution of globally rare bacterial taxa reflects environmental transitions across the surface ocean. *Mol Ecol* 2019; **28**: 1930–1945.

4. Mestre M, Ruiz-González C, Logares R, Duarte CM, Gasol JM, Sala MM. Sinking particles promote vertical connectivity in the ocean microbiome. *Proc Natl Acad Sci U S A* 2018; **115**: E6799–E6807.
